# Gastroenterology Meets Machine Learning: Status Quo and Quo Vadis

**DOI:** 10.1155/2019/1870975

**Published:** 2019-04-02

**Authors:** Amina Adadi, Safae Adadi, Mohammed Berrada

**Affiliations:** ^1^Computer and Interdisciplinary Physics Laboratory, Sidi Mohamed Ben Abdellah University, Fez 30050, Morocco; ^2^Service of Hepatology and Gastroenterology, Hassan II University Hospital of Fez, Sidi Mohamed Ben Abdellah University, Fez, Morocco

## Abstract

Machine learning has undergone a transition phase from being a pure statistical tool to being one of the main drivers of modern medicine. In gastroenterology, this technology is motivating a growing number of studies that rely on these innovative methods to deal with critical issues related to this practice. Hence, in the light of the burgeoning research on the use of machine learning in gastroenterology, a systematic review of the literature is timely. In this work, we present the results gleaned through a systematic review of prominent gastroenterology literature using machine learning techniques. Based on the analysis of 88 journal articles, we delimit the scope of application, we discuss current limitations including bias, lack of transparency, accountability, and data availability, and we put forward future avenues.

## 1. Introduction

In recent years, machine learning (ML) has got the bulk of attention. Powered by an influx of big data and advancements in computing power and coupled with considerable enthusiasm in the mainstream media, this exciting technology is driving major industry transformations. According to the statistics, a quarter of organizations worldwide are spending more than 15 % of their IT budget on ML (https://www.statista.com/statistics/695582/worldwide-machine-learning-share-of-budget/), while the global ML market is expected to reach US$ 39.98 billion by 2025 (https://www.researchandmarkets.com/research/xf4j28/the_global?w=4).

In a nutshell, ML encompasses a broad set of techniques inspired by human learning and reasoning systems; they share the same basic functioning, that is, to establish the extent to which the past is likely to be an accurate guide to the future. Endowed with this faculty of learning, these techniques are capable of analyzing large amounts of data, extracting (that is, learning) information from them, and driving automatic decisions. In this vein, ML methods are particularly suited for analyzing medical data, given their complexity, high dimensionality, and incompleteness. Therefore, in medical context, ML methods hold the potential of assisting clinicians in diseases diagnosis and prediction, thus improving delivery of quality and personalized care to patients and ultimately realizing performance improvement and efficiency gains. Like any technology, ML is not without its limitations. Security, privacy, data quality, and transparency are major concerns that hinder the use of ML in clinical settings. However, considering the increased research effort in both ML and medicine, the mix of these two areas is worth further exploring and developing.

Against this backdrop, valuable benefits are expected from the use of ML in gastroenterology (GE). Indeed, given the high prevalence and related mortality rate of digestive diseases and the amount of data generated by procedures used in this domain, addressing GE issues by mean of ML will widen diagnostic and therapeutic capabilities and beget new procedures, thereby making the scope and practice of GE more diverse, interesting, and gratifying.

The confluence of ML's immense potential with the challenges posed in GE settings has inspired a growing body of GE research relying substantially on ML. However, while the number of empirical studies concerning the use of ML for GE needs has increased, the literature lacks an overview of the extant research. Indeed, very few works discuss the topic from the literature lens. Moreover, existing works suffer from two limitations, either they are not updated [1] or they deal only with some specific issues [2]; hence lack in terms of holism, actualization, and completeness is clearly observed. By contrast, in other medical domains, such as radiology [3], oncology [4], neurosurgery [5], ophthalmology [6], diabetology [7], and orthopaedics [8], ML is obtaining a crescent interest in form of dedicated recent systematic reviews. We believe that, given the efforts made so far, it is also important for GE to evaluate the present state-of-the-art regarding the use of ML in this practice in order to chart a path toward promising and suitable directions for future research. In this respect, we carried out a systematic literature review of ML application on GE. The present work synthesizes the trends observed through the analysis of 88 papers selected from the screening of 2768 studies. We tried to make this review accessible to both gastroenterologists and ML researchers hoping that this will inspire more collaborations between the two communities and motivate the design of novel ML approaches for GE applications.

The remaining sections of this paper summarize the results of our systematic literature review as follows: in order to establish the context of this review, Section 2 proposes a preliminary background that describes key concepts of ML and addresses this technology in the broader context of medicine with a more specific focus on GE practice. In Section 3, we outline the protocol we use for our systematic literature review based on established guidelines. In Section 4, findings are reported from two perspectives, medical and technological, and supported by several illustrative charts.  Section 5 discusses research directions and open issues that we gathered from the surveyed literature. Finally, Section 6 concludes this review.

## 2. Background

This section provides an overview of the area under study, ML and its application to GE.

### 2.1. Machine Learning Overview

The literature on ML is so extensive that even a superficial overview of all the main ML approaches goes far beyond the possibilities of this paper. In this section, our aim is instead to provide the reader with some basic insights that might help better understand the remaining parts of this article.

ML is a core multidisciplinary subfield of artificial intelligence (AI) that got its inspiration from a variety of academic disciplines, including computer science, statistics, philosophy, biology, and psychology. The core function of ML is to discover patterns in data that lead to actionable insights. It includes a broad class of algorithms that share the capability of learning from previous experience to improve future performance. More precisely, following the Mitchell's definition [9], “A computer program is said to learn from experience E with respect to some class of tasks T and performance measure P, if its performance at tasks in T, as measured by P, improves with experience E”. If we suppose, for example, that the learning task is to predict hepatitis C infection after liver transplantation, the experience would be a database including data about patients who have undergone this procedure and the infection status as determined by human experts, and the performance would be the difference between real and predicted infections.

In contrast to traditional algorithms, ML algorithms do not need to be explicitly programmed to perform tasks [10]. Instead, they are fed by observation data that enable them to gradually learn how to solve problems by induction. For this to happen, the learning process of a ML-based system is divided into two phases: (a) the training phase, where an estimation of unknown dependencies in the system from a given dataset is done and, as a result, a model is constructed; (b) the testing phase, where the model is given test data that have no answers as input, and based on the training it received, the model will predict the answers. According to the approach used for the learning process, ML algorithms may be broken down into four main families, namely, (i) supervised, (ii) unsupervised, (iii) semisupervised, and (iv) reinforcement learning.

*Supervised ML* requires that the algorithm's possible outputs are already known and that the data used to train the model is already labelled with the correct answers. For example, in training a system to identify a specific liver tumour type, the label would be the tumour pathologic results or genomic information. The ML algorithm is exposed to enough of these labelled data to allow it to morph into a model designed to detect the type of liver tumour for a new given case. Supervised learning algorithms are useful for solving two kinds of learning tasks: (i) classification and (ii) regression. The task of classification refers to a learning process that categorizes the data into a set of finite classes. In the case of regression problems, a learning function maps the data into a real-value variable. Some of the most common supervised techniques are decision trees, K-nearest neighbors, support vector machines, and logistic regression.

In* unsupervised ML*, unlabelled training data are exposed to the algorithm with the goal of generating labels that will meaningfully organize the data. This is typically done by finding which examples are similar to each other and grouping them in clusters. On the other hand, a supervised classification algorithm learns to ascribe inputted labels to endoscopic images of some structure; for example, its unsupervised counterpart will look at inherent similarities between the images and separate them into groups accordingly, assigning its own new label to each group. This type of algorithm is mainly used for (i) clustering and (ii) association. The objective of clustering problem is to discoverer the inherent groupings in the data, while the objective of an association learning problem is to discover rules that describe large portions of data, which serve principally to reduce the dimensionality of the data. Examples of clustering algorithms include K-means, hierarchical clustering, and self-organizing map. The main algorithms used for association are principal component analysis, independent component analysis, nonnegative matrix factorization, and singular value decomposition.

*Semisupervised ML* is a technique that combines the power of both supervised and unsupervised learning. Typically, it takes advantage of the huge amount of unlabelled data to perform a classification. In many practical learning domains, there is large supply of unlabelled data but limited labelled data which can be expensive to generate. So semisupervised learning algorithms is used for the same applications as supervised learning but it uses both labelled and unlabelled data for training. Examples of semisupervised learning algorithms include self-training, generative models, graph-based algorithms, multiview algorithms.

Another kind of* ML is reinforcement learning* [11] which is based on behavioural psychology. This type of algorithms is trained to map actions to situation so that the reward or the feedback signal is maximized. It should be mentioned that algorithms from this family are not told which action to take, as in most forms of ML, but instead they must discover themselves which actions yield the most reward by trial and error. Thus, in this type of ML, the focus is shifted from pattern recognition to experience-driven sequential decision-making.

As we are reviewing studies that have tackled the application of ML methods on GE, we propose in Table 1 an overview of commonly used ML algorithms (techniques) in this practice.

Besides how a ML technique works, what is more crucial in practice is the resulting performance. Generally, to estimate the performance of an algorithm, one of three main variations of the cross-validation technique is used [12]:

- K-fold cross-validation: The dataset is partitioned into K subsets; each subset acts as the validation/test set once and the rest of the data are used for training the model. Model performance is then assessed by averaging the results attained from each subset.
- Leave-one-out cross-validation: The model is trained using the entire dataset except for one data point and the model is validated with the data point that was left out. This process is repeated until every data point has been used as the test data point.
- Random stratification: Set percentage of the dataset is randomly assigned to the training set and the remaining becomes the test set.

To quantify performance evaluation, different metrics are employed. The most common ones are described in Table 2.

Although the first ML algorithms have existed decades ago [10], it is only recently that stunning achievements have ignited interest in diverse domains. These advances are in part due to increases in computing power (processing, storage, and memory), but also owe a great deal to the huge quantities of data that are being generated in the Internet age. The major developments in ML that are exciting so much interest at the moment could not have been made without big data. Today, ML has become ubiquitous and increasingly vital for the evolution of a range of fields including social media, education, finance, manufacturing, energy, and medicine. It is mainly used to recognize complex patterns in observed data in order to automatically make decisions about patterns hidden in that data.

### 2.2. Machine Learning in Medicine

#### 2.2.1. Potential Application and Current Use of ML in Medicine

As modern medicine relies on the ever-increasing amounts of data and as ML algorithms—the data hungry technology—become better at finding patterns and making predictions that far exceed that of humans, it is clear that this technology can take medicine far beyond what it is capable of today.

In order to draw up the “big picture” of ML opportunities in medicine, we discuss the potential use of ML in this sector along three key areas, namely, (a) medical research and development, (b) clinical practice, and (c) population heath. From our standpoint, each of these areas is spanning its own spectrum but together they constitute a proper representation of the medical landscape.

*(a) Research and Development: Drug Discovery*. ML-driven automation can help to address some of the biggest challenges in medical research, particularly in preclinical applications such as drug discovery and genomic science. Indeed, drug development is a time-consuming and cost-intensive process encompassing the early stages of research, preclinical testing, clinical trials, and review and approval. Speeding up one of these steps in this long process would have big implications for the entire chain; for example, predicting the likelihood of toxicity in the earliest stages before undergoing the clinical trials would considerably save time and cost. ML techniques are the best candidate for improving drug discovery process; some of the potential use cases of ML in this industry include

- identifying potentially useful molecules to take forward through the drug development process,
- predicting potential side effects of drugs earlier,
- repurposing drugs, i.e., finding new uses for previously tested compounds, which is a much cheaper alternative to starting from scratch,
- identifying drugs that could work together as a combination for treatment,
- speeding up the design of clinical trials by automatically identifying suitable candidates as well as ensuring the correct distribution for groups of trial participants.

A further use for ML lies in gene editing, a complex process where specific alterations are made to DNA at the cellular level. Gene editing is considered as a revolutionized technique that appears to be emerging as a key tool for drug discovery ranging from target identification and validation to preclinical testing.

Another potential application of ML is genomics. Genomics are characterized by huge, complex datasets. Sophisticated tools relying on ML can then be used to analyze these datasets more quickly than human analysis would allow. This recent advance would make it possible to rapidly identify and interpret the genetic variation underlying a single patient's disease, thereby providing a window into patient-specific mechanisms that cause or contribute to disease, which could ultimately enable the 'precise' targeting of these mechanisms. Thus, ML is major driver in the development of drug in the era of precision medicine. 

*(b) Clinical Practice: Diagnosis and Prognosis*. The accurate prediction and identification of a disease constitute one of the most interesting and challenging tasks for physicians. In this sense, ML provides methods, techniques, and tools that can help to solve diagnosis and prognosis problems in a variety of medical domains, where the input is a dataset with characteristics of the subjects, and the output is the diagnosis or the prognosis of a specific disease.

Medical diagnosis reasoning has always been an important application area of computational intelligence techniques. Back to mid-70s, expert systems and model-based schemes provided mechanisms for the generation of hypotheses from patient data. In recent years, the interest has been shifted to the use of ML in computer-aided diagnosis; the major expected improvement over classical expert systems is a higher diagnostic accuracy through algorithms that will generate differential diagnoses, suggest high-value tests, and reduce overuse of testing.

Similarly, for medical prognosis, classical prognostic models were restricted to only a handful of variables, because humans must enter and tally the scores. With the use of ML, data could be drawn directly from a multitude of databases, allowing models to use thousands of rich predictor variables, which naturally will lead to better predictions. 

*(c) Population Health: Epidemic Outbreak Prediction*. ML can also be useful in monitoring and predicting epidemic outbreaks and biological attack around the world, especially in developing countries where this issue is particularly pressing considering the general lack of medical infrastructure and the limited access to treatments. By receiving training from different data sources such as satellites, previous outbreaks, weather, rainfall, and the total number of positive cases, ML-based systems can make predictions about the likelihood of an infectious disease outbreak like AIDS, malaria, influenza, or BSE. They can forecast the spread behaviours of these epidemic diseases well before they occur and hence help in controlling their impact and reducing casualties by implementing adequate countermeasures (quarantine, vaccination, medical treatment). However, it is important to mention that although a considerable amount of theory has studied the benefit of using ML for epidemic outbreak prediction and medicine in general, actual proven clinical performance and utilization of ML are still mostly lacking.

As a result, given its ability to deal with large, complex data and its emphasis on obtaining accurate predictions, ML can help physicians more efficiently diagnosing diseases, developing drugs, personalizing treatments, and even editing genes. Aside from those core applications, ML can also help in scenarios in which current IT solutions may not be optimal such as scheduling patients and staffing optimization, billing and collections, and patient-facing applications. However, though diagnosis and prognosis are relatively straightforward ML problems, clinical decision-making using ML is not yet widely used by the medical community. Indeed, a light scan of literature related to the current ML applications in medicine shows that there is uneven use of ML across medicine domains and that, in general, the overall work in this area is still in its infancy. To better illustrate this point, we present in Table 3 a selection of studies and applications of ML across a range of medical domains.

As depicted in Table 3, current ML use for diagnosis and prognosis needs is groundbreaking. This use is evolving increasingly in radiology, pathology, and oncology. The concentration around these specialties is not completely unexpected. All the three of them are inherently data interpretation professions, especially radiology, which deals almost exclusively with data in the form of images. The potential for pattern recognition and automated analyses of images in radiology is unique, and that is why a large number of works in literature are studying ML in this domain. Oncology and pathology are akin to radiology; the benefits seen in radiological image analysis have been translated into histological images and a number of studies applying this ability in interesting ways. Digital pathology is one of the most active fields in ML applications. Computer-aided diagnosis using ML techniques has extensively been used for analyzing and interpreting digital whole slide images (WSI) [14]. In this regard, popular ML applications for diagnosis-related tasks in digital pathology include segmentation of region of interest (ROI), scoring of immunostaining, cancer staging, and content based image retrieval [15].

Besides the three major specialties, ML has been applied in other medical domains as well. Two interesting examples are cardiology and neurology. Cardiology applications mainly deal with early detection of cardiovascular diseases; in this vein a very recent use of ML lies in monitoring heart disease. Typically, the person uses an “app” on their smartphone to measure their pulse, and a ML model detects their arterial stiffness [16]. In neurology, in addition to usual applications of brain disorders diseases detection and neuroimaging analysis, ML can enable a “physiological augmented intelligence”, a form of AI that extends human abilities. An illustrative example is a study that proposes a ML-based system capable of reading cortical activity directly from the brain, transmitting signals from a paralyzed human's motor cortex to hand muscles and restoring motor control [17]. Much more upcoming ambitious intelligent technologies like the very recent “neural lace” [18] hold the promise to revolutionize the relationship between the human brain and computer.

Furthermore, it is worth noting that the most used ML techniques are supervised including Bayesian Networks, SVM, DT, and an increasing use of DL especially in image analysis.

Most recently, we have seen ML emerging in other medical specialties that are not yet as ripe for ML as the discussed ones but surely hide an untapped potential. Most notably, otolaryngology [19], dermatology [20], endocrinology (diabetology) [21], and of course gastroenterology are no exception.

#### 2.2.2. ML in Gastroenterology

GE focuses on the digestive system and its disorders. Gastrointestinal disorders are extremely common in the general population. This makes gastroenterology an important branch of medicine since all physicians, regardless of specialty, encounter GE symptoms and diseases. This domain is concerned with prevention, investigation, and treatment of and research into illnesses involving the gastrointestinal tract and liver. GE is divided into two main subdomains, namely, hepatology and “hollow organ” gastroenterology.

Considering the presented discussion on ML potential in different medicine domains, it is expected for GE, like the other fields, to be a fertile ground for ML. Endoscopic imaging and polyps identification and differentiation among other applications are natural targets for optimization and automation via ML.

In this sense, a particular excitement has been generated by economic (industrial) players with commercial solutions and products aimed at involving ML-based systems in GE practice and procedures. Most popular examples include ai4gi (https://ai4gi.com/) that offers a package of solutions that use DL for gastrointestinal screening, Crospon (https://www.crospon.com/) that has developed EndoFLIP, a popular ML-based imaging solution for the functional assessment of the gastrointestinal tract, and a Poland-based startup cta.ai (http://cta.ai/en/projects/gastro-view) that proposes ML software called GastroView that can analyze video of the gastrointestinal tract taken with tiny cameras swallowed in the form of a capsule.

By focusing so far on ML, we discussed how this trendy technology impacts medicine in general and GE in particular. By shifting the focus to GE, it seems appropriate to cast insights on the most important technological advances in the GE space, discussing how they are connected and how ML is positioned among them.

As a matter of fact, GE is largely regarded as a technology-driven field. Indeed, in the era of the fourth industrial revolution, a number of technologies are taking strides toward transforming GE practice by providing tools to augment and extend the effectiveness of gastroenterologists. At frontline are three key enabling technologies: (a) Internet of Things (IoT) [36], (b) big data [36], and (c) AI.

*IoT*: an IoT system connects all the available medical resources as a network to perform clinical activities over the Internet. IoT devices include wearable, portable, or digestible (bio)sensors, which are connected to cloud-based platforms that help to store and analyze the captured data. They are wildly integrated with smartphones due to portability and ubiquitous availability of mobile technology. IoT opens up the possibility of* telemedicine* (monitoring patients in their homes); this way of delivering healthcare is particularly useful for chronic diseases and, thanks to IoT, it is becoming a mainstream healthcare model.

*Big data*: the driver behind all the IoT sensors is the data that is generated. Big medical data has three characteristics: it is available in extraordinarily high volume; it moves at high velocity and spans the healthcare industry's massive digital universe; and, because it derives from many sources, it is highly variable in structure and nature. This is known as the 3Vs of big data. Given its volume and availability, one of the most exciting implications of big data in medicine is enabling data-driven clinical activities with much more precise and personalized care. This goal is known as* precision medicine*.

*AI*: AI allows converting medical big data to useful and actionable information. It offers intelligent systems able to sense the world, comprehend, act, and learn. In medicine, AI is essentially considered as a* decision*-*support systems*. In addition to ML techniques, AI provides other tools including robotic surgical systems, conversational AI (chatbots), and human brain interfaces that speed up the transition to the* digital health*.

**Figure 1:**
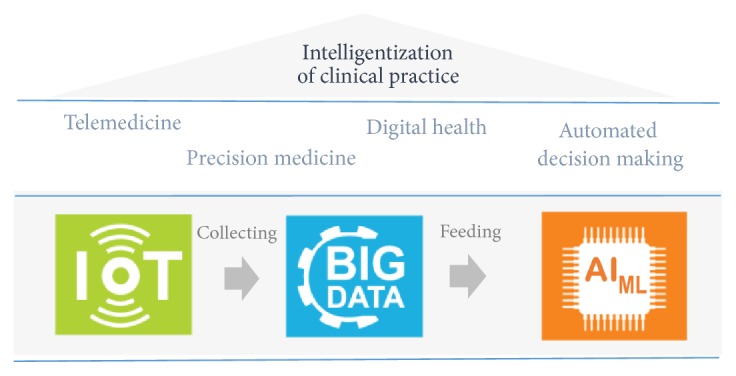
Key enabling technologies in GE practice.

It is clear that the combination of IoT, big data, and AI is much obvious and beneficial. Synergistically, each technology adds value to the other and drives the need for the other. Ample data is generated from IoT sources to feed the algorithms that find patterns in data leading to breakthroughs in therapies and treatment. Ultimately, this momentum enables the “intelligentization” of the clinical practice (Figure 1). Innovation enabled by these triplet technologies can be illustrated in practice with a typical gastroenterological scenario where belly sensors measure stomach activity while the patient is carrying out his normal daily activities, and store the generated data in a secure cloud platform on which the patient's electronic health record and other information such as laboratory results and medical and prescription histories are also stored. Based on a ML algorithm feeding by the available data, decision recommendations are provided to gastroenterologists in real time to advance the treatment.

In this chain of value, ML plays a vital role as it can be considered the brain that makes sense of available data. As a result, given the potential and current applications of ML in GE as an important branch of medicine and considering the strategic position of ML in the GE intelligentization technologies landscape, we can conclude that the meeting between ML and GE is worth further studying.

## 3. Method

This survey was carried out in a systematic manner guided by the PRISMA standards [37]. Accordingly, details about the planning and the execution of the undertaken review are giving in the following subsections.

### 3.1. Research Questions

We used the PICOC [37] model to frame our research questions:

*Population*: Interested communities including clinically oriented physicians (gastroenterologists), AI researchers, and other health professionals.

*Intervention*: ML techniques applied to GE.

*Comparison*: Not applicable. We are interested in all techniques and classifying them.

*Outcomes*: We look for ML techniques that are revolutionizing GE practice.

*Context*: Academic and clinical context, with a focus on empirical studies.

This allowed us to form the three research questions of this review described in Table 4.

### 3.2. Search Process

**Figure 2:**
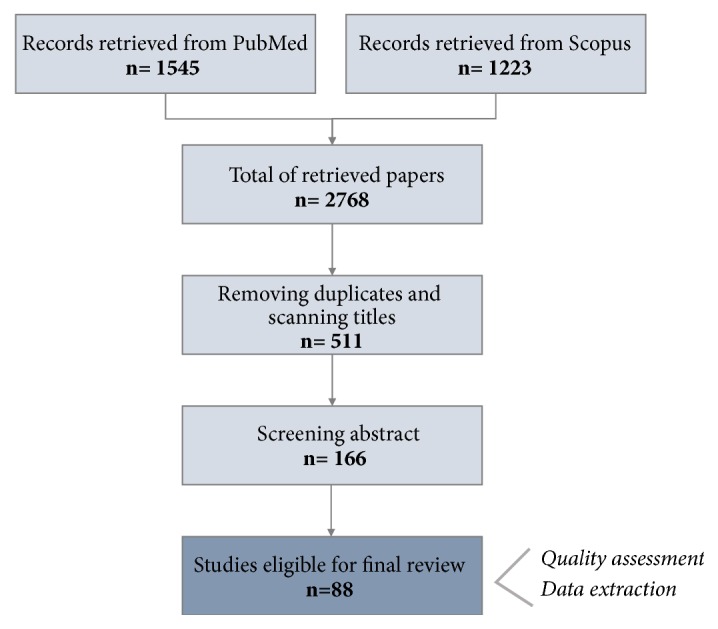
The process of search and selection of papers.

As depicted in Figure 2, the works reported herein result from a four-part search process. This involves the following steps: (i) search strategy, (ii) study selection, (iii) quality assessment, and (iv) data extraction strategy.

#### 3.2.1. Search Strategy

To select relevant works within the scope addressed in this systematic review, a query-based search was carried out in both Medline (through the Pubmed search engine) and Scopus Elsevier databases. We performed a temporally unbounded search for articles published from inception up to October 2018. We only considered studies that have been published in indexed journals and which applied at least one ML technique (MLT).

Our search strings are formed by the union of the words “*Machine Learning*” and a set of related terms including “*Artificial Intelligence*” as ML is a subfield of AI, “*Data Mining*” as in many works MLTs are mentioned as data mining techniques, “*Neural Network*”, and “*Deep Learning*”; though DL is a MLT, because of its popularity it begins to be referred to as a standalone technique; thus we considered this key-phrase explicitly. Finally, we added “*Algorithms*” as a related term, since a considerable number of medical works stay at an abstract technological level and use only the term “algorithms” to refer to MLTs (Table 5).

On the other hand, in order to capture a large range of GE studies we combined the aforementioned search strings with a set of terms related to the GE main topics of interest: Oesophagus, Stomach, Gallbladder, Liver, Pancreas, Biliary Bowel, Colon, Intestine, Gut, Anus, and Rectum, in addition to some general concepts such as Gastroenterology, Hepatology, Proctology, Endoscopy, and Digestive (Table 5).

The identified search terms were then compiled into a query using the “OR” and “AND” operators, to link, respectively, terms variation of the same group and terms of the two groups (i.e., ML and GE related terms).

#### 3.2.2. Study Selection

After removing duplicates, a first selection of the resulting papers was based on reading their titles and excluding all the irrelevant papers on the basis of this information alone. When deemed necessary, the abstract was also taken into consideration. For the selected articles, abstracts were screened. Then, available full texts were read in order to identify relevant articles based on the inclusion criteria (IC). At this stage, additional IC were applied in addition to the two aforementioned IC:

*IC1*: The study is published in indexed journal.

*IC2*: At least one MLT is used.

The following IC were also applied:

*IC3*: The full-text of the paper is accessible.

*IC4*: The paper is written in English or French.

*IC5*: GE related application is the only issue considered (we exclude general works that deal with many medical domains, for example, studies related to cancer of the liver but also that of the lung and the breast).

*IC6*: The paper presents empirically oriented work (no surveys or reviews).

*IC7*: Performance measures of the applied MLTs are clearly stated.

*IC8*: The data that were used as a source for the training and validation are reported.

As a result, 1545 articles were initially identified through Medline database searching and 1223 articles were identified through Scopus database searching (for a total of 2768 initial articles). After duplicates were removed and titles were scanned, abstracts of 511 papers were screened. Then 166 full-text articles were assessed for inclusion, with 88 studies meeting final IC. Although it is impossible to achieve a complete coverage of the literature, we believe that a significant number of relevant papers were extracted and are presented in this review.

#### 3.2.3. Quality Assessment

We assessed quantitatively the methodological quality of each selected paper based on established study quality assessment tools and guidelines [38–40]. The goal here is to assure the quality of included studies in terms of rigour, credibility, and relevance. The quality assessment questions are described below. Three possible answers could be chosen for each question, yes (Y), no (N), or partially (P). The scoring procedure was Y = 1, P = 0.5, N = 0. A study could thus score between 0 and 9. We selected the first third (i.e., 3) to act as a cutoff point, with any study scoring 3 or below being removed from the corpus. As a result of performing the quality assessment for all 88 articles, none of the included studies have got the elimination score.

 
*Question 1*: Was there a clear statement of the aims of the study?
 
*Question 2*: Was the study designed to achieve these aims?
 
*Question 3*: Was the study population clearly specified and defined?
 
*Question 4*: Are the techniques used clearly described? And is their selection justified?
 
*Question 5*: Are the data collection methods adequately detailed?
 
*Question 6*: Is the implementation process adequately detailed?
 
*Question 7*: Is there a clear statement of findings?
 
*Question 8*: Were the evaluation measures clearly defined, valid, and reliable?
 
*Question 9*: Was the conclusion supported by the reported findings?

#### 3.2.4. Data Extraction Strategy

In order to address the research questions, we examined each of the included papers in detail and extracted the following information: (a) study title, (b) date of publication, (c) country where the study was ran or the country where authors' affiliations are situated, (d) the used MLTs, (e) the type of data source, (f) the performance measures, and (g) the aim of the study (from a GE viewpoint). Then we filtered through the gathered information to find any reasonable subcategorization of the studied GE problems in terms of the application areas. After several rounds of analysis, we were able to identify five GE subfields where ML was applied and eight GE activities where ML was involved. Details about this categorization and other findings are given in the next section.

## 4. Results

### 4.1. Temporal and Regional Trends

**Figure 3:**
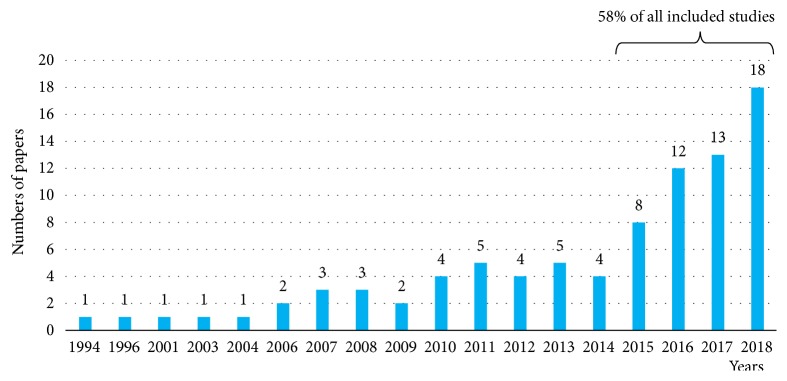
Distribution of the included studies by year of publication.

**Figure 4:**
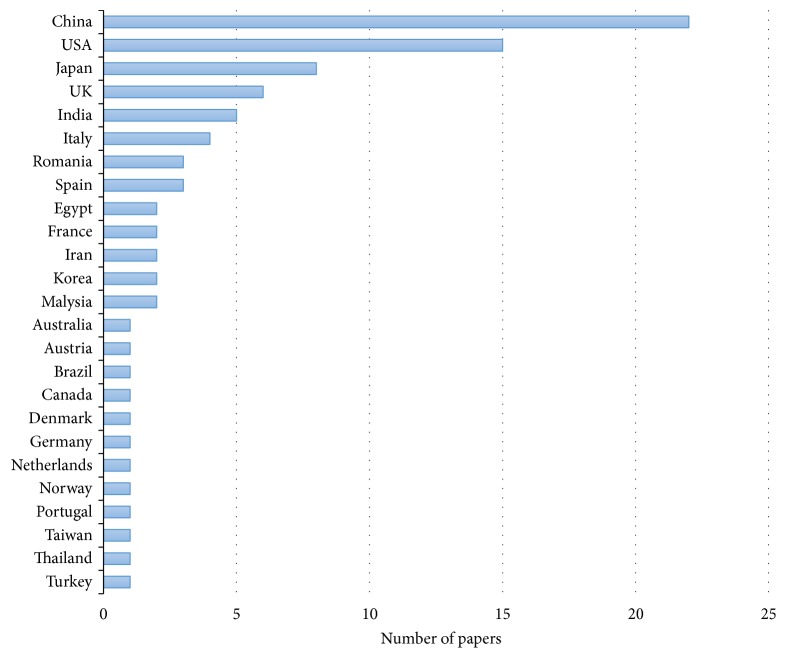
Breakdown of the included studies by country.

Figure 3 shows the distribution of the included studies by year of publication. We noted an upward trend for publications in the last four years. 58% of the reviewed studies have been published since 2015. 37.5% were published between 2004 and 2014, and only 0.5% were published before 2004. This shows that ML is becoming an increasingly common topic among GE community. We believe that this is owing to the recent democratization of the use of inexpensive and available ML tools (e.g., WEKA software, R platform, and TensorFlow framework) that can be accessible to nontechnical users. Furthermore, it should be noted that although researchers worldwide have been studying ML within GE, the leading-edge work is performed in China, USA, and Japan. The rest of the studies are mainly distributed between Europe and the UK (24 studies) and other Asian countries (14 studies). The detailed breakdown of the studies by country is provided in Figure 4.

### 4.2. GE Embraces ML: The Broad Spectrum of GE Applications

**Figure 5:**
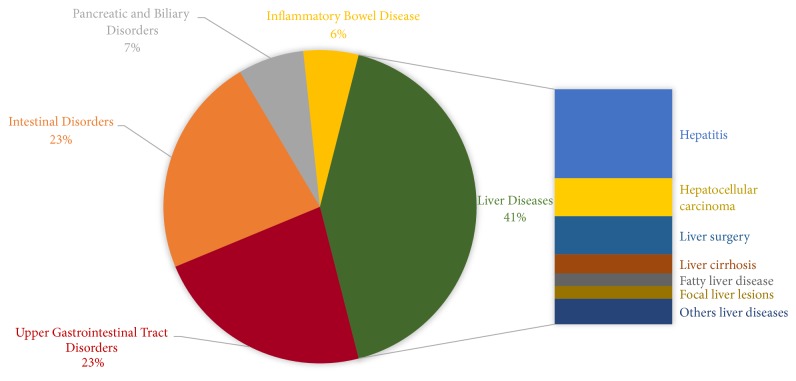
Main GE disorders covered by the identified studies.

Based on the conducted review, areas of active focus of ML within GE are related to major clinical disorders including (a) liver diseases, (b) upper gastrointestinal tract disorders, (c) intestinal disorders, (d) pancreatic and biliary disorders, and (e) inflammatory bowel disease.  Figure 5 depicts the breakdown of the reviewed studies according to the addressed disorders. Currently, a large portion of research has tackled ML from a vantage point of liver diseases (41%), although the range of applications is rapidly expanding. In this line of work, ML models have been mainly used to assist in screening for hepatitis viruses infection [S63], [S74] and liver cancer [S43], [S83], planning liver surgery [S57], [S85], and staging advanced fibrosis and cirrhosis [S51], [S82]. On the other front, considerable evidence-oriented studies have been conducted to prove the efficacy of using MLTs for diagnosing different types of gastric, oesophageal, and colorectal disorders with an innovative focus on endoscopic image analysis [S60], [S61]. Some studies have specifically dealt with inflammatory bowel disease in terms of diagnosis and risk prediction [S37], [S44]. However, less number of studies have been noted in the landscape of pancreatic and biliary disorders [S26], [S79]; this appears to be mainly due to the GE domain restriction. Indeed, since the pancreas is functioning as two glands in one: a digestive exocrine gland and a hormone-producing endocrine gland, only studies covering the digestive related functions were selected. However, it should be pointed out that “endocrine” pancreas disorders have also been the subject of several studies that demonstrate the ML potential in improving treatment and diagnosis of chronical diseases like diabetes, notably in the development of artificial pancreas [41–43].

Ultimately, ML-based approaches are used as a decision-support system to address clinical issues related to prognosis, diagnosis, treatment, and patient management. More precisely, most of the applications within GE are narrowly focused on achieving some specific tasks. An in-depth analysis of each reviewed study's aims allowed us to identify eight key tasks (activities) where MLTs assets are readily evident; Table 6 describes in detail the distilled tasks.

It should be noted that these categories of tasks have a significant amount of overlap, but they provide a useful framework for discussing current applications of ML in GE practice. Indeed, task categorization was essentially based on the primary aim of each analyzed study. However, it is clear that disease classification and accurate endoscopic images interpretation play a major role in cancer early detection and that predicting mortality, risk stratification, and outcomes prediction are important for an adapted and effective treatment planning.

**Figure 6:**
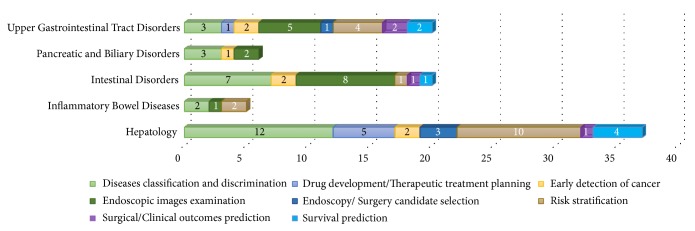
Distribution of GE activities using ML classified by GE disorders.

Figure 6 resumes the identified ML applications in GE, with an emphasis on the aiming clinical task to perform per GE practice. It stresses the clear tendency of using ML for disease classification and discrimination, endoscopic imaging examination, and risk stratification. Again, it is liver diseases area that encompasses the large range of task types in terms of scope and diversity. Although endoscopic imaging is limited in the hepatology practice, ML enabling imaging examination is discussed based on other medical imaging techniques such as multiphasic computed tomography (CT) and magnetic resonance imaging (MRI) [S15], [S82].

Besides the identification of potential improvement in major clinical use cases and practices to which ML is strongly contributing, upon further analysis, we spot studies which, by mean of ML, address some critical questions and open challenges that have widely been debated within GE community. In what follows, we outline four key issues, namely, (i) liver transplantation, (ii) hepatitis C virus infection, (iii) gastric-intestinal cancer management, and (iv) computer-aided endoscopic diagnosis. 

*(a) Liver Transplantation*. Liver transplantation (LT) is the gold standard for treatment of end-stage liver disease of various etiologies. However, this life-saving therapy involves several clinical challenges. The most controversial issue in transplantation is the large discrepancy between the increasing demand for organs and limited number of donors, which makes optimization of scarce resources a priority. In this sense, ML-based techniques have shown the potential utility of offering an efficient decision-support model to assist medical experts in determining candidacy for LT.

Doyle et al. [S1] introduced ML algorithms as reliable tools for prediction of outcomes in terms of morbidity and mortality early after liver transplantation. Dorado-Moreno et al. [S57] used ANN to predict the probability of organ survival at different thresholds for each donor-recipient pair. Following the concept of benefit of survival, Briceño et al. [S35] proposed ML-based donor-recipient matching model for an objective liver transplant.

In an effort to prognosticate recurrent hepatitis C virus (HCV) infection after LT, Piscaglia et al. [S6] and Stoean et al. [S21] adopted ML techniques to predict the presence or absence of significant fibrosis with the aim of staging HCV hepatitis in LT recipients. The former based their approach on ANN whereas the latter defended the use of SVM and genetic algorithms as an innovative and more performant techniques.

Moccia et al. [S85] investigated the automatic analysis of liver texture with semisupervised ML algorithms to automate the hepatic steatosis assessment process, which is of primary importance for lowering liver dysfunction risks after transplantation. This study is considered as the first attempt to use MLTs and automatic texture analysis of RGB images from ubiquitous smartphone cameras for the task of graft hepatic steatosis assessment.

Reviewed studies regarding LT used different sources of information ranging from clinical data, biochemical data, and medical imaging. ANN and SVM are the most used MLTs. Algorithm performance is mainly evaluated by accuracy measurement and it is often compared against current standards of donor and recipient risk assessment, such as DRI, MELD, and SOFT score [S9], [S58]. 

*(b) Hepatitis C Virus Infection*. HCV infection is a major cause of liver-related morbidity and mortality. With no vaccine and a prevalence estimated at 2.5% (177.5 million of HCV infected adults) [44], this virus is considered as a global health challenge. Hence, accurate prognosis, the rate of progression, and effective antiviral treatments of HCV represent major concerns that are preoccupying the research agenda of many GE communities. In this respect, several studies have investigated the use of MLTs in HCV diagnosis in the quest of accuracy and early diagnosis. Lara et al. [S64] proposed an ANN based model to identify acute and recent stages of HCV infection using the genetic information of hypervariable region 1 data. In an effort to improve the accuracy of hepatitis disease diagnosis, a hybrid machine learning approach was proposed by Nilashi et al. [S74] by combining the outputs of several predictors. Recently, it has been demonstrated that MLTs are very useful in exploring patterns of care; Chirikov et al. [S65] used RF to identify the quality of care patterns correlated with treatment receipt among Medicare disabled patients with HCV infection.

HCV causes an increasing level of liver-related morbidity and mortality due to the disease progression. For this reason, previous and more recent studies have focused on automated monitoring of HCV associated diseases. Kurosaki et al. [S28] used DT analysis to build a predictive model for the identification of patients at high risk of developing hepatocellular carcinoma in order to personalize the treatment plan for chronic HCV. In another line of work, hepatic fibrosis is considered the principal indicator of progressive liver disease within HCV infection. It needs to be accurately staged for an immediate antiviral therapy in case of a significant level. An obvious trend in the clinical practice at this regard consists of developing noninvasive markers of liver fibrosis as an alternative to invasive and discomforting liver biopsy. In this sense, numerous studies have shown that MLTs have a great potential to improve the noninvasive diagnosis of significant fibrosis in HCV due to their ability to discover the hidden predictive patterns from medical databases. Using different predictive models and sharing the same goal of accurately and individually predicting early stages of liver fibrosis, studies such as [S6], [S21], [S27], [S73] have proposed valid and reliable tool to assist in liver fibrosis staging.

Due to the large number of people infected, HCV is also an attractive target for the development of antiviral drugs. Weidlich et al. [S31] employed MLTs and structure-activity relationship (SAR) analysis to identify novel inhibitors of NS5B of HCV genotype 1b. They proposed a ligand-based drug design approaches in an effort to reposition known drugs as potential HCV therapeutics and to identify new chemical scaffolds for inhibitors. In the same vein, Worachartcheewan et al. [S38] explored the chemical space of a set of HCV NS5B inhibitors and performed molecular fragment analysis; they constructed a classification model using a set of quantum chemical and molecular descriptors, modeled using ML classifiers. 

*(c) Gastrointestinal Cancer Management*. Cancer is among the leading causes of death worldwide. At the intersection of oncology and gastroenterology, early detection and prognosis of gastrointestinal cancer (GI) represent a critical issue. Several studies have been reported in the literature regarding the application of ML in prognosis/prediction of different types of GI. According to the nature of the predictive tasks, reviewed studies can be divided into three main classes: 

*(i) Prediction of Cancer Susceptibility*. A. Săftoiu et al. [S26] assessed the accuracy of real-time endoscopic ultrasound elastography in focal pancreatic lesions using computer-aided diagnosis by ANN analysis. Based on their neural computing approach, pancreatic cancer has been classified correctly in 82.95% of cases. However an unbalanced distribution of pseudotumoral chronic pancreatitis and pancreatic cancer patients was observed. Sirinukunwattana et al. [S46] proposed a Spatially Constrained Convolutional Neural Network for detection and classification of cell nuclei in histopathology images of cancerous tissue. The evaluation was conducted on a large dataset with 20 000 annotated nuclei from samples of different histologic grades. The results showed that the proposed approach could potentially offer a systematic quantitative analysis of tissue morphology and tissue constituents, lending itself to be a useful tool for better understanding of the tumour. Recently, Haj-Hassan et al. [S67] have also used convolution neural networks to predict three tissue types related to the progression of colorectal cancer: benign hyperplasia, intraepithelial neoplasia, and carcinoma. An accuracy of 99.17% was obtained from segmented image regions, outperforming existing approaches based on traditional feature extraction and classification techniques. In their work, Daniel et al. [S55] demonstrated the basic principles for the breathomics to classify gastric cancer using backpropagation neural network (BPN). This work carried out a comparative study of the result obtained by the single- and multilayer cascade-forward and feed-forward BPN with different activation functions. Results showed that the multilayer cascade-forward BPN outperforms the classification of gastric cancer from normal and benign cases. 

*(ii) Prediction of Cancer Recurrence*. In their pilot study, Woolsey et al. [S84] demonstrated that a powerful predictive model for tumour recurrence can be identified by combining radiomic signatures derived from quantitative texture analysis of the* in situ* gastric tumour and normal liver parenchymal tissue when used alone or combined with clinical outcomes using two ML algorithms: random forest classification and logistic regression. Ogihara et al. [S56] introduced their study as a proof-of-concept that ML algorithms can be an invaluable tool, supporting the decision-making process for liver transplant organ allocation. They used ANN and random forest classifier to predict the likelihood of redeveloping liver cancer after liver transplantation. 

*(iii) Prediction of Cancer Survival*. Mofidi et al. [S7] proposed an ANN based method for prediction of survival from carcinoma of oesophagus and oesophago-gastric junction following surgical resection. The accuracy of the ANN in predicting survival at 1 and 3 years reached 88%, which outperforms the classical method of staging for patients with oesophageal and oesophagogastric junction carcinoma (the Union for International Cancer Control TNM classification system). Santos et al. [S43] introduced a new cluster-based oversampling method for improving survival prediction of hepatocellular carcinoma patients. The method is based on K-means clustering and the SMOTE algorithm to build a representative dataset and use it as training example for different machine learning algorithms (logistic regression and neural networks). The results are evaluated in terms of survival prediction and compared across baseline approaches that do not consider clustering and/or oversampling. The results showed that the proposed methodology coupled with neural networks outperformed all other classical approaches currently used in hepatocellular carcinoma prediction models. Peng et al. [S49] developed a scoring system based on ANN for predicting 10-year survival in stage II A colon cancer patients after radical surgery. They showed by calculating the 10-year overall survival rates and the 10-year disease free survival rates that their scoring system could help to predict long-term survival and screen out high-risk individuals for more vigorous treatment. Recently, Chaudhary et al. [S77] presented a deep learning based multiomics integration that robustly predicts survival in liver cancer. The model is recognized as the first study that employs deep learning to identify multiomics features linked to the differential survival of patients with hepatocellular carcinoma. Given its robustness over multiple cohorts, the authors expect this workflow to be useful at predicting liver cancer prognosis prediction.

Furthermore, it is worth mentioning that the 23 identified studies regarding this issue deal with four types of cancer: colorectal cancer (8 studies), liver cancer (7 studies), gastric and oesophageal cancer (6 studies), and pancreatic cancer (2 studies). The predictive models depicted in these studies are mainly based on supervised ML techniques as well as on different input features and data samples including gene expression profiles and clinical variables as well as histological parameters. 

*(d) Computer-Aided Endoscopic Diagnosis*. GE endoscopy is a major diagnostic and therapeutic tool in clinical gastroenterology; it involves, among others, making diagnosis in real time based on the visual appearances. In order to overcome limitations regarding human eye reduced accuracy in identification and characterization of polyp and intra- and interobserver variability, a computer-aided diagnosis (CAD) system based on the texture appearances of polyps is highly demanded to support diagnosis during endoscopic examinations. In this background, several studies have suggested that the use of ML-based CAD with advanced image processing and accurate pattern recognition capabilities can improve significantly the overall outcomes of endoscopic diagnosis.

Specifically, ML-based CAD systems were shown to be effective in the differentiation of malignancy/benignancy for lesions. Norton et al. [S3] and Săftoiu et al. [S11] developed a self-learning CAD that can analyze endoscopic ultrasonographic (EUS) images and differentiate pancreatic malignancy from pancreatitis. In their work, Nguyen et al. [S19] studied the role of ML-based image analysis in differentiating EUS features of potentially malignant gastrointestinal subepithelial lesions from those of benign lesions. Byrne et al. [S59] trained a ML model on endoscopic videos to differentiate diminutive adenomas from hyperplastic polyps in real time using DL.

Another major application of ML-based CAD is the capsule endoscopy (CE) imaging interpretation. Indeed, CE, as a noninvasive endoscopic modality to visualize the entire gastrointestinal tract, generates a tremendous amount of data that can effectively be analyzed by MLTs. Reviewed applications include detection of bleeding in the digestive tract [S14], [S22], [S86], polyp recognition [S24], [S52], [S61], intestinal motility assessment [S17], and hookworm detection [S76].

In another vein, in order to increase the efficient use of endoscopy, Buri et al. [S18] have used ML-based CAD as a decision-making support to select appreciate patients for upper endoscopy. Another decision-support system that identifies candidate patients with acute gastrointestinal bleeding for urgent endoscopy has been proposed by Chu et al. [S13].

While the most state-of-the-art endoscopic CAD methods require labelled data to train various supervised machine learning models, in an interesting recent study, Wang et al. [S53] designed a new CAD system without human specific labelling using multiple-instance learning technique.

Finally, the preponderance of the use of endoscopic imaging as a source of data is worth noting; more than 40% of all reviewed studies based their experiments on endoscopic data. Furthermore, ANN is, without a double, the technique of choice for treating this type of data with a remarkable emergence of DL technique in recent studies [S48], [S59], [S75], [S72], [S80].

### 4.3. ML Penetrates GE: The Broad Portfolio of Learning Techniques

**Figure 7:**
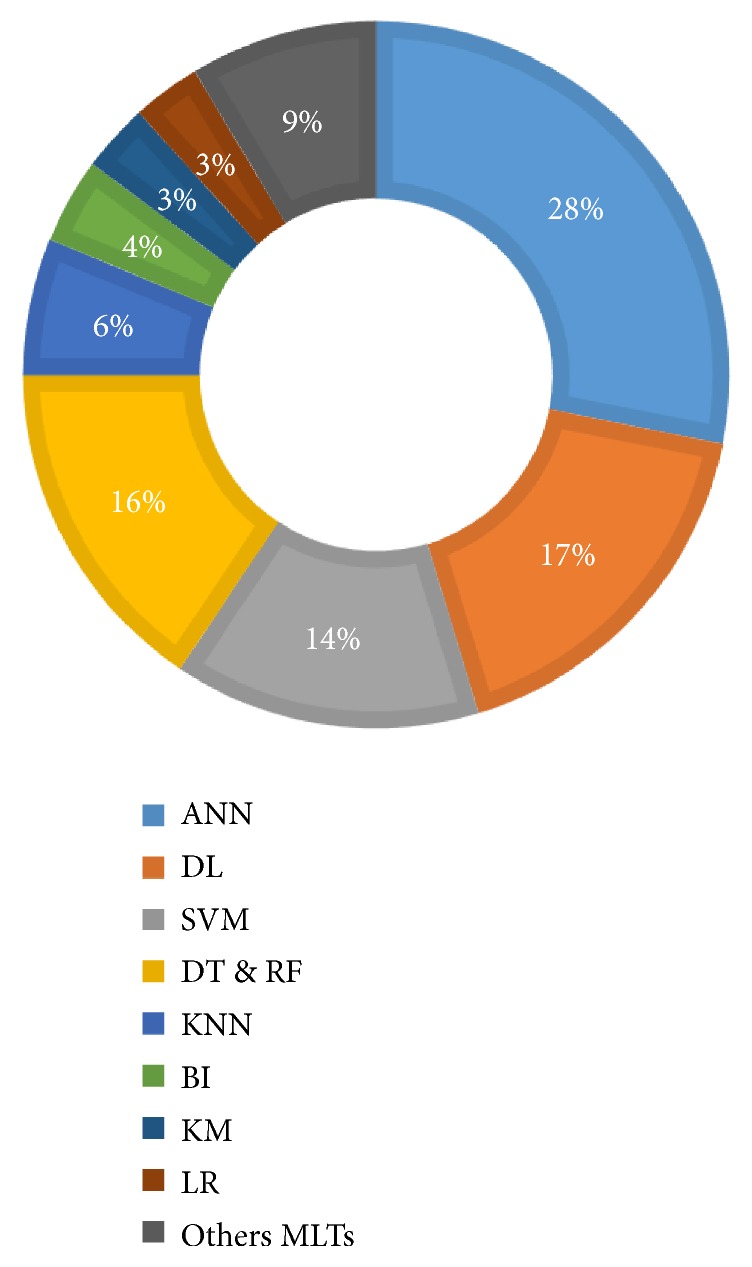
The main used MLTs.

**Figure 8:**
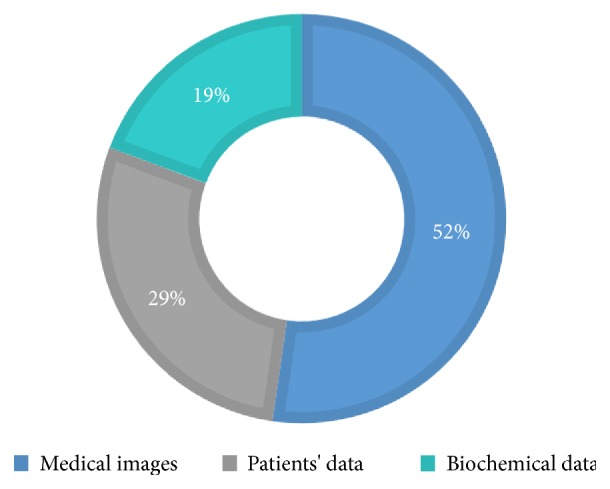
Data source typology.

**Figure 9:**
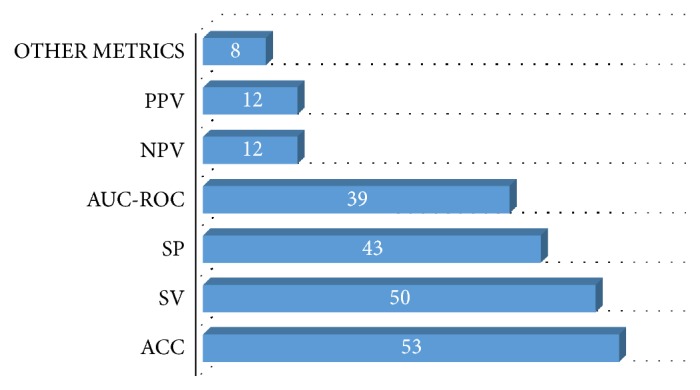
The main used performance metrics.

**Figure 10:**
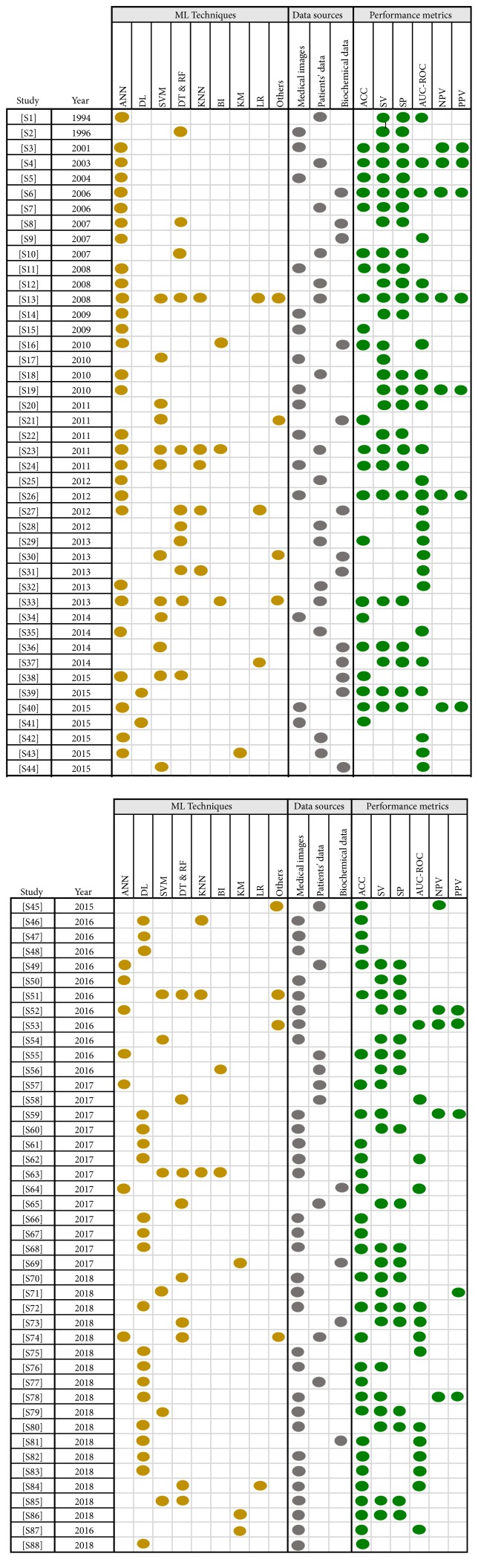
Technical description of all included studies.

Reviewed studies follow a typical design that includes the application of one or more* ML algorithms* to a* set of data* in order to perform a clinical task with a certain level of* performance*. Hence, the scientific relevance and interest of a given study are strongly associated with three key elements: (1) the used MLT, (2) the typology of data, and (3) the achieved performance. Next, we present the result of analyzing the 88 studies from these three-dimension standpoint. We support our findings by three illustrations describing the level of use of each MLT (Figure 7), the data source typology (Figure 8), and the performance measures (Figure 9). The technical description of all the included studies is given in Figure 10.

#### 4.3.1. ML Algorithms

Depending on how MLTs are used, studies can be divided into three classes:

- *Studies using a single MLT*: Generally, this class of studies introduce the use of a specific MLT for a given clinical problem and prove its usefulness by measuring its performance [S59].
- *Studies using multiple MLTs working in complementary fashion*: This range of studies deal with a complex clinical problem that requires the use of a composition of MLTs that work synergistically to perform the overall task [S74].
- *Studies using multiple MLTs working in competition manner*: These include works aimed at optimization by identifying the best model that outperform the state-of-the-art techniques. Typically, these types of studies carry out comparison of performance using some references like ROC curves [S13].

Across studies, we identified the use of a plethora of MLTs for different objectives (Figure 7). Unarguably, ANN is the most used MLT as 28% of identified studies have adopted this technique. Early studies [S1], [S3], [S4], [S5], [S6], which used ANN in its basic form, suggested that this technique is well suited for tasks like clinical diagnosis and surgery/clinical outcomes prediction and that it may perform better than other traditional approaches like LR and classical expert systems. Nevertheless, they noted a limitation regarding the quantity of training and testing datasets that represents an influential parameter affecting the model performance. In the era of big data, recent studies described ANN as a complex and flexible nonlinear systems with unique properties including robust performance in dealing with noisy or incomplete input patterns, high fault tolerance, and the capability to describe interactions between risk factors. Thus, recently, ANN has increasingly been used in predicting survival [S42], [S49], [S57], [S64].

Furthermore, the analysis of recent literature regarding the use of ML in GE practice confirms that this field is broadly in line with the recent surge of interest in DL. Indeed, the use of DL has grown exponentially since 2015 with 22 identified studies in just the last four years. Considered as a go-to model on medical image pattern recognition related problems, the implementation of DL in GE does not surprisingly involve imaging. Several works have explored DL, especially through CNNs, to enable the extraction of highly representative features. This is done among the network layers by filtering, selecting, and using these features in the last fully connected layers for pattern recognition. However, this performance is achieved at the cost of transparency. Application examples of DL include 3D CT images segmentation [S41], [S66], [S82], [S83], real-time assessment of endoscopic images [S59], [S62], [S72], [S80], capsule endoscopy image/video analysis [S60], [S61], [S76], and classification of histology images [S46].

In the last decade, a growing trend is noted in the use of other supervised learning techniques, namely, SVM and DT. SVM has generated much enthusiasm because of its high discriminative power (14% of studies). This technique is designed for high-dimensional data with a large feature space (large number of predictor variables) compared to the sample size. Thus, it has been used to solve clinical problems such as mining liver fibrosis [S21] and risk prediction for inflammatory bowel disease [S30] and as a CAD for difficult-to-diagnose diseases like early detection neoplastic lesions in Barrett's esophagus [S54] and celiac disease diagnosis [S23]. However, though the accuracy of prediction deriving from SVM is reported to be superior to that resulting from other MLTs [S13], the complexity of the traditional mathematical treatment of the inherent optimization task is somewhat uninviting [S21].

On the other hand, DT has been described through literature as a preferred algorithm for building understandable predictive models, that is simple yet fast-to-build, with good accuracy and easy conversion to classification rules. Studies that have adopted DT to build their models [S2] [S10], [S28], [S70], [S73] agree on the simplicity of this technique as it does not require any domain knowledge and it is easy to assimilate by physicians. RF, a derived DT technique with better predictive accuracy, has also been widely used as predictive model [S29], [S58], [S65]. However, while RF results in more reliable predictions than single trees, it is difficult to interpret, as individual trees are lost in the overall forest [S65].

Other techniques have yet been less successfully used in a range of GE applications. Notably, KNN (8 studies), an attractive technique with its simplicity and its ability to predict complex nonlinear behaviour, has been combined with other techniques to achieve better accuracy [S27], [S31], [S46], [S63]. By introducing a probabilistic treatment, Bayesian inference (BI) (5 studies) is reported to be better in overcoming problems related to local trapping, overfitting, and overtime in training. It is even proposed to have significant advantages over the conventional neural network approach [S16], [S33], [S63], [S56]. KM (4 studies) has been used as a clustering method for applications like liver vessel segmentation [S86] and bleeding frame and region detection [S87].

The use of classical techniques like LR [S13], [S37], [S84] has also been observed in the literature, serving mainly performance comparison needs. Finally, the rest of studies (9%) have introduced other techniques including gradient boosting [S45], rule induction [S33], nearest shrunken centroid [S13], multiple-instance learning [S53], self-organizing map [S74], genetic algorithm [S21], principal component analysis (PCA) [S74], and linear discriminant analysis [S13], [S51].

#### 4.3.2. Data Sources

Looking back to the previous decade, only molecular information and clinical information were exploited for making predictions/diagnosis. With the rapid development of high throughput technologies including genomic, proteomic, and imaging technologies, new types of input data source have been collected. As depicted in Figure 8, there are three types of data that were mainly used to train and apply MLTs, namely, medical imaging (46 studies), patients' data (25 studies), and biochemical data (17 studies).

Medical imaging prevails as the main data source, which comes as no surprise, since imaging is an area that can naturally take advantage of the pattern recognition capabilities of MLTs. This includes interpretation of endoscopic images/videos which encompass a large variety of image sources such as capsule endoscopy [S60], [S87], endoscopic ultrasound elastography [S19], [S26], colonoscopy [S59], [S62], chromoendoscopy [S71], and esophagogastroduodenoscopy [S75]. Besides endoscopic imaging, several studies proposed ML approaches to analyze CT images [S41], [S84], histology images [S34], [S46], MR imaging [S15], ultrasound images [S40], [S88], and images provided by real-time tissue elastography, the emerging ultrasound imaging technology [S63].

Furthermore, typical information that has been used as a data source in the analyzed studies is patients' data. Indeed, the integration of features such as symptoms, family history, age, diet, demographic data, and high-risk habits surely plays a critical role in predicting and diagnosing GE disorders [S12], [S18], [S28]. However, as claimed by some studies' authors [S13], these types of parameters do not provide sufficient information for making robust decisions. In this sense, biochemical data have been proven as very informative indicators for disorder detection and prognosis [S9], [S38], [S39], [S69]. Based on microarray technology, some works studied whether miRNA expression data, in conjunction with MLT, is suitable as a noninvasive test for major predictive and diagnosis decision [S44], [S81]. A noted trend in the reviewed literature includes the integration of mixed data, such as clinical and genomic data [S77]. Other examples include studies that have been conducted to discover the interaction between FibroScan stiffness indicator, biochemical data, and clinical examinations toward a respective degree of liver fibrosis [S21], [S27], [S73]. Furthermore, smartphones and wearable technology along with electronic patient records have notably been used to collect all types of data [S85].

#### 4.3.3. Performance Evaluation

Validation of the proposed system is an inescapable step that concluded each reviewed study. Indeed, in order for the proposed approaches to be used in the real clinical setting, their performance must be evaluated. In the surveyed literature, various forms of cross-validation have been applied and different evaluation metrics have been used.

Based on the chart presented in Figure 9, among the evaluation metrics mentioned in the literature, accuracy has extensively been used (53 studies have used accuracy or other measures that can be equated to accuracy), followed by specificity and sensitivity (respectively, 50 and 43 studies), which are by the way common measures in medical literature. ROC curve analysis was used in 39 studies to evaluate the performance. This latter was also assessed in terms of the positive predictive values and negative predictive values in 12 studies.

Generally, these evaluation metrics estimate performance in different ways. Thus, it is important to choose ones that are consistent with the target domain. In the GE setting, it is true that only predictive performance related measures are considered (other measures like speed, scalability, and interpretability are not used). However, it is not always clear in the reviewed studies why a predictive performance measure was used over another. Mostly, in order to underpin their proposal, researchers used the above measures together (15% of the studies used more than 4 common metrics mentioned above).

Other measures, which are mentioned much less frequently, encompass likelihood ratios for positive and negative tests, discriminant power value, Confidence Interval, Matthews correlation coefficient, F-measures, and Dice Similarity Coefficient, which is used to validate the spatial overlap accuracy in automatic image segmentation tasks.

Even though it is difficult to comprehensively compare different techniques under different conditions. It is worth noting that by examining the reported models' performance, the achieved accuracy ranged from 80% to 99% according to the solved problem, data sizing, and the used technique. The highest accuracy of 99.17% goes for cancer early detection using CNN to build a decision-support system for classifications of multispectral colorectal cancer tissues [S67]. As noted before, this has no deep signification. Indeed, to conduct a fair comparison, the problem and the used data should be the same for all researches to be able to compare different applied MLTs.

#### 4.3.4. Learning Problems

Similarly to the narrative review proposed in the previous section regarding the major GE issues addressed in the reviewed literature. In what follows, we describe works that dealt with fundamental learning problems. The idea is to provide a complete balanced analysis of literature in a way to advocate the beneficial link between GE and ML.

Accordingly, we report studies aimed at developing models focusing on three main learning problems: (a) classification, (b) clustering and segmentation, and (c) dimensionality reduction. 

*(a) Classification*. The majority of studies (~80%) applied supervised prediction and classification algorithms capable of modeling linear and nonlinear relationships between variables to develop predictive/diagnosis models. In general, classifier studies trained a MLT using a labelled dataset (e.g., endoscopic images associated with known outcomes) to iteratively evaluate, compare, and select variables that would determinate with the highest accuracy the class of the observation (e.g., normal/benign cases for cancer or none/early and advanced fibrosis/cirrhosis for liver fibrosis degree). When a new observation is received (unlabelled dataset), it is classified based on previous experiences.

Various classification techniques were used in different clinical tasks and for several GE disorders particularly for diseases classification and discrimination, risk stratification, and survival prediction.

Studies for diseases classification and discrimination include classification of gastric cancer using backpropagation ANN [S55], classification of white regions in liver biopsies by SVM [S34], differentiation between pancreatic malignancy and pancreatitis using ANN [S11], and differentiation of adenomatous and hyperplastic diminutive colorectal polyp using DL [S59].

For risk stratification, examples of studies include predicting outcomes in patients with perforated gastroduodenal ulcers using ANN [S42], predicting advanced liver fibrosis in chronic hepatitis C using DT [S73], and predicting risk for inflammatory bowel disease using SVM [S30].

Lastly, classification-learning problem has been studied in predicting survival in liver cancer using DL [S77], in predicting 10-year survival in stage II A colon cancer patients after radical surgery using ANN [S49] and in a SVM model for predicting mortality in gastric cancer [S20]. 

*(b) Clustering and Segmentation*. Conversely to the previous range of works, unsupervised machine learning models were applied on an unlabelled dataset for clustering or segmentation needs.

Aiming to improve survival prediction of hepatocellular carcinoma patients, Santos et al. [S43] proposed a cluster-based oversampling approach that is robust to small and imbalanced datasets. KM technique has been used to assess the underlying patient groups in the studied dataset. The results were evaluated in terms of survival prediction and compared across baseline approaches that do not consider clustering and/or oversampling.

Zeng et al. [S86] proposed an automatic method for liver vessel segmentation based on the application 3D region growing and hybrid active contour model combined with K-means clustering for thick vessel segmentation. They showed that their method is capable of segmenting complex liver vessels with more continuous and complete thin vessel details, outperforming several existing 3D vessel segmentation algorithms.

Based on CT images, Gayathri et al. [S41] and Hu et al. [S47] used, respectively, ANN and DL for an automatic 3D segmentation of organs characterized by complex backgrounds, ambiguous boundaries, heterogeneous appearances, and highly varied shapes. Hwang et al. focused on the advantages of CAD techniques for colon segmentation, which aid in the identification of polyps for the detection of colorectal cancer with an accuracy of 98%, while Hu et al. focused on liver segmentation as a fundamental task in computer-assisted liver surgery planning. 

*(c) Dimensionality Reduction*. Clinical data involved in diagnostic/predictive models are usually high dimensional. High-dimensional datasets increase the complexity of classification and reduce the effect of models. Thus, before building models, some studies proposed tools to reduce the data dimension while retaining essential information of the original data.

Chu et al. [S13] used RF and SVM for high-dimensional data reduction. They argued that in addition to being good classifiers, these MLTs are particularly relevant for the high dimensionality problem in the context of prediction source and severity of acute gastrointestinal bleeding, as its dataset is characterized by a large number of predictor variables.

A SVM based model was proposed by Stoean et al. [S21] for staging liver fibrosis in chronic hepatitis C. The application of PCA, a commonly used feature extraction mechanism, has led to a sizeable reduction of the data dimensionality from 24 to only 6 assembled attributes. However, a decrease in the achieved accuracy was observed. The presented method was, thus, endowed with a mechanism for dynamic feature selection provided by a genetic algorithm that achieved a good result in terms of dimensionality reduction without impacting the model accuracy.

Acharya et al. [S51] proposed an automated characterization of fatty liver disease and cirrhosis using curvelet transform method and entropy features extracted from ultrasound images. The dimensionality of the extracted feature was reduced using discriminant analysis. Then the discriminant analysis coefficients ranked based on F-value were fed to different classifiers to choose the best performing classifier using a minimum number of features. The proposed method has attained 97.33% accuracy, 96% sensitivity, and 100% specificity using ANN classifier based on only six features.

Finally, it should be noted that some studies dealt with issues that involve all the aforementioned learning problems. For instance, Nilashi et al. [S74] proposed a hybrid ML approach for hepatitis disease diagnosis using self-organizing map technique for the clustering of data in the experimental dataset, PCA for reducing dimensionality and improving the accuracy of clustering, DT as a supervised learning technique for the selection of the most important features, and adaptive neurofuzzy inference system technique (a type of ANN) for hepatitis disease diagnosis.

## 5. Discussion

In this work, we reviewed publications in which ML approaches were applied in GE practice. To the best of our knowledge, this is the most comprehensive systematic review regarding the topic. For the sake of completeness and in order to answer our research questions we reported our results from two complementary perspectives: (a) the impact of ML in GE; in this regard we discuss how this technology is revolutionizing the field, notably by providing innovative solutions toward solving challenging GE issues; (b) the dynamic of ML in GE by focusing, this time, on the diversity of the used algorithms, the quality of their predictive performance, and the typology of the employed data sources.

In doing so, we made sure to present first a synthesis of findings in form of statistics and charts and subsequently we gave more insights about some studies. For the sake of synthesis and relevance, not all the surveyed studies were explicitly detailed in this work. Our informal criteria for this choice are as follows: (a) the papers are deemed to be a significant work (received high citation level) and (b) they pertinently address and illustrate the discussed issues/problems. In addition, for each issue/problem, we tried to cover the whole corresponding timeline by citing references dating back to 1994 to have an overview of the progress made so far.

In what follows, we present a compilation of the main findings along with a discussion of some research directions and open problems distilled from the surveyed works.

### 5.1. Current and Potential GE Applications

We observed that a wide range of GE areas was related, to a greater or lesser degree, to potential applications of ML. Particularly, liver diseases have been extensively addressed; this can be attributed to the medical pressure generated by the liver‐related mortality and morbidity. Indeed, the prevalence of hepatocellular carcinoma and hepatitis infection indicates that the burden of chronic and neoplastic liver diseases is substantial and translates into a significant public health problem. This makes hepatology related practices an area of opportunity for ML to prove its effectiveness in terms of prevention, diagnosis, and therapy of patients with liver diseases.

However, this opportunity can also potentially be seen in other GE areas that seem to be underexplored by researchers, for instance, nutrition disorders. Even though recently clinical nutrition has been very much welcomed into the family of GE, in our review we did not encounter relevant studies that discuss this type of disorders. We believe that early diagnosis and/or prediction of nutritional disorders such as anorexia, obesity, malabsorption, and anemia by means of ML is a potential application of this technology and that more focus should be given to this area. More studies are also expected in the neurogastroenterology field; as an advancing subspecialty of GE dealing with neurological relations to functional gastrointestinal disorders, this area can benefit from contributions done in both GE and neurology fields. Recently, some works have begun to study the opportunity of another promising application of ML that is related to intestinal microbiome [45, 46]. Indeed, studies have shown that the knowledge of the gut microbiome and the interrelationships with the human body bring major opportunities for diagnosis, prognosis, and treatment of a variety of human diseases. In this vein, MLTs can efficiently be used for automatic extraction of knowledge from the large amounts of data produced by the research of the human microbiome.

Furthermore, as noted in the previous section, thanks to ML, a multitude of complex clinical tasks that were exclusively done by physicians before have been automated. This includes planning surgery and predicting its outcomes but not direct gastrointestinal surgery related tasks. In fact, by conceiving our research query (Section 3) we purposely focused on clinical GE, while also not totally disregarding surgical procedures. Indeed, principally surgery practice involves two broad areas: surgical decision‐making and operative surgery. While the first area was covered in this work (choices about the need for surgery, timing of surgery, potential risks, and the likelihood of mortality), we believe that innovation in the second area will be essentially derived by medical robotics [47], another powerful tool of AI.

Ultimately, the use of ML in GE practice aims to provide the ability to support decisions and to reduce the number of invasive tests for reliable prognosis and diagnosis. This continuous penetration of ML in everyday medical practice had gradually pushed the general debate about AI/ML taking away the need for a human workforce to the realm of the medical community. According to this claim, because AI-based systems outperform their human counterparts in tasks involving analyzing large volumes of data and finding patterns, they are becoming indispensable and vital tools in the physician's arsenal and slowly the role of this latter is eroding. Most surveyed studies refute this extremist vision. Since human experts and machines have different strengths, they advocated the use of ML as a complement rather than a replacement of human experts. ML can serve as an ideal “second opinion”; however, the final decision will always be made by physicians.

Finally, across studies, it was confirmed that personalized treatment/precision medicine is deeply connected to and dependent on the use of ML. Indeed, several studies have shown [S56], [S21], [S27] how ML approaches can lead to medical decisions based on individual patient characteristics which allow customizing and tailoring the treatment plan to an individual's unique disease.

### 5.2. Trends in the Use of MLTs

A large portfolio of MLTs was employed in the reviewed studies with an increasing attention to DL in recent years. Some studies used unsupervised learning algorithms while the majority of studies used supervised one. However, there are no reports on the use of reinforcement learning (RL). In the literature of other medical fields, there exist several examples of RL applications, which have been used to develop treatment strategies for epilepsy [48] lung cancer [49] and in developing artificial pancreas [50]. Practically, RL includes problems where an agent attempts to improve its performance at a given task over time by continual interaction with its environment. Thus, it is suited to problems including sequences of decisions along a timeline. Additionally, it can be used when decisions depend on the observed state. In this view, we believe that personalized treatment plans and nutrition disorders are particularly well suited for RL application in GE setting. Nutrition disorder effects are unpredictable, making it necessary to closely monitor the patient's condition. For personalized treatment, RL can offer an attractive alternative to classical treatment systems that are based exclusively on the current state of patients, by taking into account not only the immediate effect of treatment, but also the long-term benefit to the patient. Hence, using reinforcement algorithms that incorporate deep learning (deep reinforcement learning) can enhance the aforementioned potentialities of RL with the powerful predictive ability of DL.

Furthermore, it has been observed that the choice of the use of some MLTs over others is generally poorly justified. There is no guidance on which would work best in one situation or another. A common rule of thumb is to try a number of algorithms and compare their performance with the aim of finding the most optimal one [S13], [S23], [S33], [S51]. However, this is obviously time and cost consuming especially if some approaches are unlikely to work well a priori. As mentioned before, unfortunately this is also the case of the evaluation metrics that are used in an additive way; that is, in order to prove the quality of some algorithms in given settings, we should use as much metrics as possible. Comparison across studies is thus not possible in absence of a consensus regarding the manner in which such models are constructed and applied or their results are objectively evaluated. We believe that more research efforts should be done in this sense in order to establish a solid theatrical background to guide and support the end-to-end process of ML approaches implementation. Apart from this, it is worth mentioning that in order to strengthen their models, some studies use a kind of MLT composition which includes a mix of algorithms (supervised and unsupervised) that are interplaying in respect of some process in order to reach a complex objective [S47], [S74]. The advantage conferred by this composition is seen in the high performance reached by the overall model.

### 5.3. Acceptance in Clinical Setting

Another fundamental observation is that in spite of the number of studies that have been published during the past two decades, very few have actually penetrated the “real-world” clinical setting. From our standpoint, this can be attributed to three limitations: 

*(a) Lack of Validation for Clinical Setting*. Despite the impressive high level of performance reported in the studies, this performance remains questionable as the proposed models are deployed in the research environment only; the same models may behave differently when deployed in a “real-world” situation and poorly generalize to other populations and regions. Thus, for an algorithm to be established in clinical setting rigorous validation is required. This includes internal and external validation and validation in a prospective clinical trial. This process is time-consuming and costly. Consequently, the number of clinically validated studies is limited in the literature. Therefore, optimal validation model should be developed in order to capitalize on the intensive research efforts made so far and to ensure a faithful translation into the clinical setting. 

*(b) Difficulties in Interpretation*. While MLTs can detect complex patterns in large data and yield accurate predictions, due to their black-box nature they are unable to provide an explanation of their outcomes. Moreover, there is often a tradeoff between accuracy and interpretability: the most accurate ML models usually are not very explainable (e.g., DL, RF, and SVM), and the most interpretable models usually are less accurate (e.g., logistic regression and DT). Explainability is a crucial issue though; it is very important to understand the decision process of a predictive model before its decision can be utilized in clinical setting since it affects the life and death of a patient. Technically, explainable AI (XAI) [51], the research field that aims to make AI/ML systems results more understandable to humans, is still in its incipient phase. Considerable interesting works have already been done, especially in domains involving life-threatening decisions such as self-driving vehicles, military, and healthcare. However, hearty research effort is yet to be expected.

Another related issue is the problem of accountability. Who is responsible for a medical error if the diagnosis is provided by a black-box AI where no doctor was involved? This shed light on one of AI's biggest modern problems, trust and ethics. Indeed, intensive use of AI/ML raises natural questions about the distribution of responsibility and authority and the role of moral values and principles in decision-making. Again, this issue is of greater consequence in medicine because decisions are literally a matter of life and death. One of the leading initiatives responding to this issue is recognized as responsible AI [52], an emerging research field concerned with incorporating ethics in AI/ML systems at design, production, use, and governance of AI. In this sense, we think that insights from bioethics can help in developing such responsible models for healthcare. Implementing adapted regulations plays also a major role in governing the interaction of medical ecosystem actors, namely, patients, doctors, and AI. 

*(c) Data Quality and Quantity*. Some of the reasons for slow acceptance in clinical setting may relate to data. As has been noted in many of the studies cited in the previous section, it is challenging to find large unbiased sources of data due to lack of public datasets. Consequently, the lack of data to feed ML models can result in misclassifications and risks “overfitting” the data with loss of generalizability. Except the data size, another limitation concerns imbalanced data, that is, when the number of observations belonging to one class is significantly lower than those belonging to the other classes. This happens because ML models are usually designed to optimize the overall accuracy without considering the relative distribution of each class. However, this could bias the MLT toward the majority class and thus affects the overall performance. Technically speaking, data augmentation [53] and transfer learning [53] approaches appear to be promising solutions for small dataset. Some researchers have also introduced crowd-sourcing as a viable alternative approach for medical data collection [54]. Nevertheless, still availability and free dissemination of data are the primary prerequisites for progress in the clinical application of ML.

Lastly, it should be emphasized that the limited number of gastroenterologists currently trained in ML methods, the lack of collaboration between physicians and ML scientists, and limited financial investments to install ML tools are also factors that hinder the penetration of ML in the GE clinical setting.

As a result, we can conclude that the current body of research exploring the utility of ML in the GE is very promising, in the sense that it proposes a set of tools to augment and extend the effectiveness of gastroenterologists in numerous domains and diverse practices. However, the implementation in clinical realm is limited by the lack of clinical validation, quality, and availability of data and by the inability of AI to display some human characteristics like being explainable and ethical.

## 6. Conclusion

In this work, a systematic effort was made to identify and review ML approaches applied to GE practice. Against a comprehensive background that covered all aspects related to ML and its application in medicine in general and in GE in particular, synthesis of reviewing 88 studies was presented. Findings showed that ML is yielding promising potential to provide valuable assistance to gastroenterologists in their main tasks. However, while the promise of ML is taking shape, there is still a gap between its potential and its effective usability in clinical settings. In order to improve clinical acceptance of ML systems, thought should be given to unravelling the “black-box” nature of MLTs, to establishing more validation models for clinical environment, and to making medical data available and freely disseminated. Furthermore, more ML application in some promising but underexplored areas is expected in the near future, notably in nutrition disorders, neurogastroenterology, and gut microbiome. This notwithstanding, considering the speed with which advances are made in the breadth and depth of ML applications, this exciting technology is expected to significantly influence the current and future practice of GE.

## Figures and Tables

**Table 1:** Overview of ML techniques.

Algorithm	Overview
Artificial neural networks (ANN)	ANN is inspired by interconnections between neurons in biological neural networks. It consists of a set of nodes configured in layers (input, hidden, and output), connected to one another via weighted edges. Input feature vectors are processed sequentially by every layer in the net via non-linear transformations, before an output is generated upon reaching the final layer. During the training process, if the output of the ANN is incorrect, an algorithm known as backpropagation distributes the error term back up through the layers, by modifying the weights at each edge. ANN can be supervised or unsupervised. More recently, there has been a resurgence of interest in multi-layered ANNs or Deep Learning (DL), given their ability to work well with complex and high-dimensional data sets. Convolutional Neural Network (CNN), a variation of DL, is a useful technique used in image classification.

Support vector machine (SVM)	SVM is a discriminative classifier formally defined by a separating hyperplane. In other words, given labelled training data, the algorithm outputs an optimal hyperplane that categorizes new examples.

Decision Tree (DT)	DT is the simplest tree-based supervised ML model. The aim is to recursively construct a tree structure, in which each internal node represents a condition based on which the tree splits into branches/ edges. The end of the branch that does not split anymore is the decision/leaf. Importantly, trees can be combined using ensemble learning to yield potent classifiers such as Random Forests (RF) and Boosted Trees.

k-Nearest neighbours (KNN)	KNN is supervised algorithm that classifies new data by a majority vote of its neighbors, with the data being assigned to the class most common amongst its K nearest neighbors measured by a distance function.

Logistic regression (LR)	LR is a traditional statistical method for solving binary classification problems (problems with two class values). It predicts the probability of occurrence of an event by fitting data to a logistic function.

K-mean clustering (KM)	KM is a popular unsupervised ML algorithm. The algorithm works iteratively to partition data into k clusters in which each object belongs to the cluster with the nearest mean. This technique produces exactly k different clusters of greatest possible distinction. The best number of clusters k leading to the greatest separation (distance) is not known a priori and must be computed from the data.

**Table 2:** Overview of performance metrics.

Metric	Formula	Description
Sensitivity (SV)	$\frac{TP}{TP+FN}$	It measures the portion of positives that are correctly identified (performance measure of the whole positive of a dataset)

Specificity (SP)	$\frac{TN}{TN+\;FP}$	It measures the portion negatives that are correctly identified (performance measure of the whole negative part of a dataset)

Positive Predictive Value (PPV)	$\frac{TP}{TP+FP}$	The ratio of correctly diagnosed positives to the total of identified positives

Negative Predictive Value (NPV)	$\frac{TN}{TN+FN}$	The ratio of correctly diagnosed negatives to the total of identified negatives

Accuracy (ACC)	$\frac{TP+TN}{TP\;+FP+TN\;+FN}$	The ratio of correctly diagnosed cases to the total diagnosed cases ( the overall performance measure)

Area under the receiver operating characteristics curve (AUC-ROC)	Graphical plot [13]	In a Receiver Operating Characteristics (ROC) curve the sensitivity is plotted in function of the false positive rate (100-Specificity) for different cut-off points of a parameter. Each point on the ROC curve represents a sensitivity/specificity pair corresponding to a particular decision threshold. The area under the ROC curve (AUC-ROC) is a measure of how well a parameter can distinguish between two diagnostic groups (diseased/normal)

TP: true positive (number of positive cases correctly detected).

TN: true negative (number of negative cases correctly detected).

FP: false positive (number of negative cases incorrectly detected as positive).

FN: false negative (number of positive cases incorrectly detected as negative).

**Table 3:** ML applications in medical domains.

Medicine domain	ML applications	References
Radiology	Radiological imaging tasks such as: (i) Risk stratification. (ii) Therapy response. (iii) Lesions segmentation and classification. (iv) Multi-omics disease discovery. (v) Discovery of radiographic imaging biomarkers. (vi) Creating study protocols.	[22–26]

Pathology	Digital pathological image analysis notably: (i) Tissue phenomics. (ii) Histopathological imaging analysis. (iii) Whole Slide imaging analysis.	[14, 27, 28]

Oncology	Early cancer diagnosis and prognosis: (i) Cancer metastases detection. (ii) Molecular subtyping of cancer. (iii) Cancer detection from microarray gene expression data (iv) Risk classification of cancer survival.	[29–31]

Cardiology	Early detection of cardiovascular diseases based on: (i) Electrocardiographic interpretation. (ii) Echocardiography interpretation. (iii) Myocardial perfusion analysis. (iv) Discrimination of different diseases with similar symptoms like constrictive pericarditis and restrictive cardiomyopathy or hypertrophic cardiomyopathy and physiological hypertrophy.	[32–34]

Neurology	Neurological disorders identification and prediction: (i) Electroencephalography data interpretation (ii) Electromyography data interpretation (iii) Augmented Intelligence such as: (iv) Restoring the control of movement in patients with quadriplegia. (v) Controlling upper-limb prostheses via Brain-computer interface.	[17, 35]

**Table 4:** Research questions.

Research question	Objective
RQ1: What are the main machine learning techniques that have been applied on gastroenterology?	Identifying techniques currently in use and studying their characteristics and outcomes in terms of learning class, sources of data, and performance.

RQ2: Which sub-fields of gastroenterology has machine learning been applied to?	Identifying where ML is making changes and hence identifying the potentially fruitful GE application domains that are still unexplored.

RQ3: How ML will impact gastroenterology practice?	Drawing conclusions about the current research efforts and the main research directions

**Table 5:** Search terms used for query.

ML related terms	GE related terms
Artificial Intelligence – Machine learning – Data mining – Neural network – Deep learning – Algorithms	Oesophagus – Stomach – Gallbladder – Liver –Pancreas – Biliary bowel – Colon – Intestine – Anus – Gut – Rectum– Gastroenterology – Hepatology – Proctology – Endoscopy – Digestive

**Table 6:** GE activities using ML.

Aim of study	Number of studies	Application/Description
Disease classification and discrimination	27	The usage of ML in disease classification is very frequent. Indeed, as ML systems are capable to analyze large volumes of patient data, they can, efficiently and accurately, correlate these features with some disease state. This is particularly useful for difficult-to-diagnose diseases, such as celiac disease which involves multiple clinical presentations and symptoms shared with other diseases. ML ability to accurately classify disease states (present/absent), etiology, and subtype allows subsequent investigations, treatments, and interventions to be delivered in an efficient and targeted manner.

Risk stratification	17	The accurate assessment of a patient's risk of adverse events remains a mainstay of clinical care; MLTs form an attractive platform to build risk metrics because they can easily incorporate disparate pieces of data, yielding classifiers with improved performance.

Endoscopic imaging examination	16	Endoscopic procedures generate a large amount of images in one examination of a patient. It is hard for clinicians to leave continuous time to examine the full endoscopic images. Thus, the use of ML to assist in endoscopic imaging examination tasks represents a response to the urgent need for new technologies to supplement existing imaging techniques.

Early detection of cancer	7	Early identification of cancer is challenging because symptoms are non-specific (or absent) and compounded by overlap with symptoms of other diseases. That is why ML has emerged as a promising technique for handling complex interactions of high-dimensional medical data related to cancerology tasks.

Survival prediction	7	Survival probability prediction is one important problem encountered in medical studies when the primary endpoint of interest is time to an event. An accurate survival probability prediction can provide a useful tool for selecting prevention and treatment strategies. Thus, considerable studies in the reviewed literature have introduced MLTs as a rapid and reliable technique to predict survival.

Others tasks	14	Other applications of MLTs that have been studied in literature with promising results include drug development and treatment planning (6 studies), endoscopy or surgery candidate selection (4 studies), and surgical/clinical outcomes prediction (4 studies).

## Supplementary Materials

Supplementary Materials A list of the 88 studies that have been selected and reviewed.
