# Supplementary material for: Gastroenterology Meets Machine Learning: Status Quo and Quo Vadis
Source: Adv Bioinformatics. 2019 Apr 2;2019:1870975. doi: 10.1155/2019/1870975 (PMC6466966; doi:10.1155/2019/1870975)
Supplement: Supplementary Materials — A list of the 88 studies that have been selected and reviewed. [file 1870975.f1.docx]

**Supplementary List S1:** Selected SLR list

[S1] HR.Doyle, I.Dvorchik, S.Mitchell, IR.Marino, FH.Ebert, J.McMichael, JJ.Fung, Predicting outcomes after liver transplantation. A connectionist approach, Ann Surg, 219(4),1994, pp.408-415.

[S2] WZ.Liu, AP.White, MT.Hallissey, JW.Fielding, Machine learning techniques in early screening for gastric and oesophageal cancer, Artif Intell Med, 8(4),1996, pp.327-341.

[S3] ID.Norton, Y.Zheng, MS.Wiersema, J.Greenleaf, JE.Clain, EP.Dimagno, Neural network analysis of EUS images to differentiate between pancreatic malignancy and pancreatitis, Gastrointest Endosc, 54(5),2001, pp.625-629.

[S4] R.Banerjee, A.Das, UC.Ghoshal, M.Sinha, Predicting mortality in patients with cirrhosis of liver with application of neural network technology, J Gastroenterol Hepatol, 18(9),2003, pp.1054-1060.

[S5] CR.Huang, BS.Sheu, PC.Chung, HB.Yang, Computerized diagnosis of Helicobacter pylori infection and associated gastric inflammation from endoscopic images by refined feature selection using a neural network, Endoscopy, 36(7),2004, pp.601-608.

[S6] F.Piscaglia, A.Cucchetti, S.Benlloch, M.Vivarelli, J.Berenguer, L.Bolondi, AD.Pinna, M.Berenguer, Prediction of significant fibrosis in hepatitis C virus infected liver transplant recipients by artificial neural network analysis of clinical factors, Eur J Gastroenterol Hepatol, 18(12),2006, pp.1255-1261.

[S7] R.Mofidi, C.Deans, MD.Duff, AC.de Beaux, S.Paterson Brown, Prediction of survival from carcinoma of oesophagus and oesophago-gastric junction following surgical resection using an artificial neural network, Eur J Surg Oncol, 32(5),2006, pp.533-539.

[S8] JM.Luk, BY.Lam, NP.Lee, DW.Ho, PC.Sham, L.Chen, J.Peng, X.Leng, PJ.Day, ST.Fan , Artificial neural networks and decision tree model analysis of liver cancer proteomes, Biochem Biophys Res Commun, 361(1),2007, pp.68-73.

[S9] A.Cucchetti, M.Vivarelli, ND.Heaton, S.Phillips, F.Piscaglia, L.Bolondi, G.La Barba, MR.Foxton, M.Rela, J.O'Grady, AD.Pinna, Artificial neural network is superior to MELD in predicting mortality of patients with end-stage liver disease, Gut, 56(2),2007, pp.253-258.

[S10] F.Firouzi, M.Rashidi, S.Hashemi, M.Kangavari, A.Bahari, NE.Daryani, MM.Emam, N.Naderi, HM.Shalmani, A.Farnood, M.Zali, A decision tree-based approach for determining low bone mineral density in inflammatory bowel disease using WEKA software, Eur J Gastroenterol Hepatol, 19(12),2007, pp.1075-1081.

[S11] A.Săftoiu, P.Vilmann, F.Gorunescu, D.Gheonea, M.Gorunescu, T.Ciurea, GL.Popescu, A.Iordache, H.Hassan, S.M.Iordache, Neural network analysis of dynamic sequences of EUS elastography used for the differential diagnosis of chronic pancreatitis and pancreatic cancer, Gastrointest Endosc, 68(6),2008, pp.1086-1094.

[S12] A.Das, T.Ben-Menachem, FT.Farooq, GS.Cooper, A.Chak, MV.Sivak, RC.Wong, Artificial neural network as a predictive instrument in patients with acute nonvariceal upper gastrointestinal hemorrhage, Gastroenterology, 134(1),2008, pp.65-74.

[S13] A. Chu, H.Ahn, B.Halwan, B.Kalmin, EL.Artifon, A.Barkun, MG.Lagoudakis, A.Kumar, A decision support system to facilitate management of patients with acute gastrointestinal bleeding, Artif Intell Med, 42(3),2008, pp.247-259.

[S14] G.Pan, G.Yan, X.Song, X.A.Qiu, BP neural network classification for bleeding detection in wireless capsule endoscopy, J Med Eng Technol, 33(7),2009, pp.575-581.

[S15] X.Zhang, M.Kanematsu, H.Fujita, X.Zhou, T.Hara, R.Yokoyama, H.Hoshi, Application of an artificial neural network to the computer-aided differentiation of focal liver disease in MR imaging, Radiol Phys Technol, 2(2),2009, pp.175-182.

[S16] D.Wang , Q.Wang, F.Shan, B.Liu , C.Lu , Identification of the risk for liver fibrosis on CHB patients using an artificial neural network based on routine and serum markers, BMC Infect Dis, 10(1),2010, pp.1-8.

[S17] F.Vilarino, P.Spyridonos, F.Deiorio, J.Vitria, F.Azpiroz, P.H.Radeva, Intestinal motility assessment with video capsule endoscopy: automatic annotation of phasic intestinal contractions, IEEE Trans Med Imaging, 29(2),2010, pp.246-259.

[S18] L.Buri, C.Hassan, G.Bersani, M.Anti, MA.Bianco, L.Cipolletta, E.Di Giulio, G.Di Matteo, L.Familiari, L.Ficano, P.Loriga, S.Morini, V.Pietropaolo, A.Zambelli, E.Grossi, M.Intraligi, M.Buscema, Appropriateness guidelines and predictive rules to select patients for upper endoscopy: a nationwide multicenter study, Am J Gastroenterol, 105(6),2010, pp.1327-1337.

[S19] VX.Nguyen, CC.Nguyen, B.Li, A.Das, Digital image analysis is a useful adjunct to endoscopic ultrasonographic diagnosis of subepithelial lesions of the gastrointestinal tract, J Ultrasound Med, 29(9),2010, pp.1345-1351.

[S20] XP.Zhang, ZL.Wang, L.Tang, YS.Sun, K.Cao, Y.Gao, Support vector machine model for diagnosis of lymph node metastasis in gastric cancer with multidetector computed tomography: a preliminary study, BMC Cancer, 11(1),2011, pp.1-6.

[S21] R.Stoean, C.Stoean, M.Lupsor, H.Stefanescu, R.Badea, Evolutionary-driven support vector machines for determining the degree of liver fibrosis in chronic hepatitis C, Artif Intell Med, 51(1),2011, pp.53-65.

[S22] G.Pan, G.Yan, X.Qiu, J.Cui, Bleeding detection in Wireless Capsule Endoscopy based on Probabilistic Neural Network, J Med Syst, 35(6),2011, pp.1477-1484.

[S23] JM. Tenório, AD.Hummel, FM.Cohrs, VL.Sdepanian, IT.Pisa, H.de Fátima Marin, Artificial intelligence techniques applied to the development of a decision-support system for diagnosing celiac disease, Int J Med Inform, 80(11),2011, pp.793-802.

[S24] B.Li, MQ. Meng, JY.Lau , Computer-aided small bowel tumor detection for capsule endoscopy, Artif Intell Med, 52(1),2011, pp.11-16.

[S25] A.Biglarian, E.Bakhshi, MR.Gohari, R.Khodabakhshi, Artificial neural network for prediction of distant metastasis in colorectal cancer, Asian Pac J Cancer Prev, 13(3),2012, pp.927-30.

[S26] A.Săftoiu, P.Vilmann, F.Gorunescu, J.Janssen, M.Hocke, M.Larsen, J.Iglesias-Garcia, P.Arcidiacono, U.Will, M.Giovannini, CF.Dietrich, R.Havre, C.Gheorghe, C.McKay, DI.Gheonea, T.Ciurea , Efficacy of an artificial neural network-based approach to endoscopic ultrasound elastography in diagnosis of focal pancreatic masses, Clin Gastroenterol Hepatol, 10(1),2012, pp.84-90.

[S27] AM.Hashem, ME.Rasmy, KM.Wahba, OG.Shaker, Single stage and multistage classification models for the prediction of liver fibrosis degree in patients with chronic hepatitis C infection, Comput Methods Programs Biomed, 105(3),2012, pp.194-209.

[S28] M.Kurosaki, N.Hiramatsu, M.Sakamoto, Y.Suzuki, M.Iwasaki, A.Tamori, K.Matsuura, S.Kakinuma, F.Sugauchi, N.Sakamoto, M.Nakagawa, N.Izumi, Data mining model using simple and readily available factors could identify patients at high risk for hepatocellular carcinoma in chronic hepatitis C, J Hepatol, 56(3),2012, pp.602-608.

[S29] AG.Singal, A.Mukherjee, BJ.Elmunzer, PD.Higgins, AS.Lok, J.Zhu, JA.Marrero, AK.Waljee, Machine learning algorithms outperform conventional regression models in predicting development of hepatocellular carcinoma, Am J Gastroenterol, 108(11),2013, pp.1723-1730.

[S30] Z.Wei, W.Wang, J.Bradfield, J.Li, C.Cardinale, E.Frackelton, C.Kim, F.Mentch, K.Van Steen, PM.Visscher, RN.Baldassano , H.Hakonarson, Large sample size, wide variant spectrum, and advanced machine-learning technique boost risk prediction for inflammatory bowel disease, Am J Hum Genet, 92(6),2013, pp.1008-1012.

[S31] IE. Weidlich, IV.Filippov, J.Brown, N.Kaushik-Basu, R.Krishnan, MC.Nicklaus, IF.Thorpe , Inhibitors for the hepatitis C virus RNA polymerase explored by SAR with advanced machine learning methods, Bioorg Med Chem, 21(11),2013, pp.3127-3137.

[S32] MH. Zheng, KQ.Shi, XF.Lin, DD.Xiao, LL.Chen, WY.Liu, YC.Fan, YP.Chen, A model to predict 3-month mortality risk of acute-on-chronic hepatitis B liver failure using artificial neural network, J Viral Hepat, 20(4),2013, pp.248-255.

[S33] Y.Kumar, G.Sahoo, Prediction of different types of liver diseases using rule based classification model, Technol Health Care, 21(5),2013, pp.417-432.

[S34] S.Vanderbeck, J.Bockhorst, R.Komorowski, DE.Kleiner, S.Gawrieh, Automatic classification of white regions in liver biopsies by supervised machine learning, Hum Pathol, 45(4),2014, pp.785-792.

[S35] J.Briceño, M.Cruz-Ramírez, M.Prieto, M.Navasa, J.Ortiz de Urbina, R.Orti, M.Gómez-Bravo, A.Otero, E.Varo, S.Tomé, G.Clemente, R.Bañares, R.Bárcena, V.Cuervas-Mons, G.Solórzano, C.Vinaixa, Z.Rubín, J.Colmenero, A.Valdivieso A5, R.Ciria, C.Hervás-Martínez, M.de la Mata, Use of artificial intelligence as an innovative donor-recipient matching model for liver transplantation: results from a multicenter Spanish study, J Hepatol, 61(5),2014, pp.1020-1028.

[S36] N.Wang, Y.Cao, W.Song, K.He, T.Li, J.Wang, B.Xu, HY.Si, CJ.Hu, AL.Li, Serum peptide pattern that differentially diagnoses hepatitis B virus-related hepatocellular carcinoma from liver cirrhosis, J Gastroenterol Hepatol, 29(7),2014, pp.1544-1550.

[S37] RP.Arasaradnam, E.Westenbrink, MJ.McFarlane, R.Harbord, S.Chambers, N.O'Connell, C.Bailey, CU.Nwokolo, KD.Bardhan, R.Savage, JA.Covington, Differentiating coeliac disease from irritable bowel syndrome by urinary volatile organic compound analysis--a pilot study, PLoS One, 9(10),2014, pp. e107312.

[S38] A.Worachartcheewan, V.Prachayasittikul, N.Anuwongcharoen, W.Shoombuatong, V.Prachayasittikul, C.Nantasenamat, On the Origins of Hepatitis C Virus NS5B Polymerase Inhibitory Activity Using Machine Learning Approaches, Curr Top Med Chem, 15(18),2015, pp.1814-1826.

[S39] Y.Xu, Z.Dai, F.Chen, S.Gao, J.Pei, L.Lai, Deep Learning for Drug-Induced Liver Injury, J Chem Inf Model, 55(10),2015, pp.2085-2093.

[S40] YN.Hwang, JH.Lee, GY.Kim, YY.Jiang, SM.Kim, Classification of focal liver lesions on ultrasound images by extracting hybrid textural features and using an artificial neural network, Biomed Mater Eng, 26(S1),2015, pp.S1599-S1611.

[S41] K.Gayathri Devi, R.Radhakrishnan, Automatic segmentation of colon in 3D CT images and removal of opacified fluid using cascade feed forward neural network, Comput Math Methods Med, 2015(1),2015.

[S42] K.Søreide, K.Thorsen, JA. Søreide, Predicting outcomes in patients with perforated gastroduodenal ulcers: artificial neural network modelling indicates a highly complex disease, Eur J Trauma Emerg Surg, 41(1),2015, pp.91-98.

[S43] MS. Santos, PH.Abreu, PJ.García-Laencina, A.Simão, A.Carvalho, A new cluster-based oversampling method for improving survival prediction of hepatocellular carcinoma patients, J Biomed Inform, 58 (2015),2015, pp.49-59.

[S44] M. Hübenthal, G.Hemmrich-Stanisak, F.Degenhardt, S.Szymczak, Z.Du, A.Elsharawy, A.Keller, S.Schreiber, M.Franke AHübenthal, G.Hemmrich-Stanisak, F.Degenhardt, S.Szymczak, Z.Du, A.Elsharawy, A.Keller, S.Schreiber, A.Franke, Sparse Modeling Reveals miRNA Signatures for Diagnostics of Inflammatory Bowel Disease, PLoS One, 10(10),2015.

[S45] L.Ayaru, PP.Ypsilantis, A.Nanapragasam, RC.Choi, A.Thillanathan, L.Min-Ho, G.Montana, Prediction of Outcome in Acute Lower Gastrointestinal Bleeding Using Gradient Boosting, PLoS One, 10(7),2015.

[S46] K.Sirinukunwattana, S.Ahmed Raza, T.Yee-Wah, DR.Snead, IA.Cree, NM.Rajpoot, Locality Sensitive Deep Learning for Detection and Classification of Nuclei in Routine Colon Cancer Histology Images, IEEE Trans Med Imaging, 35(5),2016, pp.1196-1206.

[S47] P.Hu, F.Wu, J.Peng, P.Liang, D.Kong, Automatic 3D liver segmentation based on deep learning and globally optimized surface evolution, Phys Med Biol, 61(24),2016, pp.8676-8698.

[S48] E.Ribeiro, A.Uhl, G.Wimmer, M.Häfner, Exploring Deep Learning and Transfer Learning for Colonic Polyp Classification, Comput Math Methods Med, 2016 (1),2016.

[S49] JH.Peng, YJ. Fang, CX.Li, QJ.Ou, W.Jiang, SX.Lu, ZH.Lu, PX.Li, JP.Yun, RX.Zhang, ZZ.Pan, S.Wan, A scoring system based on artificial neural network for predicting 10-year survival in stage II A colon cancer patients after radical surgery, Oncotarget, 7(16),2016, pp.22939-22947.

[S50] M.Ozkan, M.Cakiroglu, O.Kocaman, M1.Kurt, B.Yilmaz, G.Can, Korkmaz, E.Dandil, Z.Eksi, Age-based computer-aided diagnosis approach for pancreatic cancer on endoscopic ultrasound images, Endosc Ultrasound, 5(2),2016, pp.101-107.

[S51] UR.Acharya, U.Raghavendra, H.Fujita, Y.Hagiwara, JE.Koh, T.Jen Hong, VK.Sudarshan, A.Vijayananthan, CH.Yeong, A.Gudigar, KH.Ng, Automated characterization of fatty liver disease and cirrhosis using curvelet transform and entropy features extracted from ultrasound images, Comput Biol Med, 79 (2016), pp.250-258.

[S52] AF.Constantinescu, M.Ionescu M, VF.Iovănescu, ME.Ciurea, AG.Ionescu, CT.Streba, MG.Bunescu, I.Rogoveanu, CC.Vere, A computer-aided diagnostic system for intestinal polyps identified by wireless capsule endoscopy, Rom J Morphol Embryol, 57(3),2016, pp.979-984.

[S53] S.Wang, Y.Cong, H.Fan, L.Liu, X.Li, Y.Yang, Y.Tang, H.Zhao, H.Yu, Computer-Aided Endoscopic Diagnosis Without Human-Specific Labeling, IEEE Trans Biomed Eng, 63(11),2016, pp.2347-2358.

[S54] F.van der Sommen, S.Zinger, WL.Curvers, R.Bisschops, O.Pech, BL.Weusten, JJ.Bergman, PH.de With, EJ.Schoon, Computer-aided detection of early neoplastic lesions in Barrett's esophagus, Endoscopy, 48(7),2016, pp.617-624.

[S55] DA.Daniel, K.Thangavel, Breathomics for Gastric Cancer Classification Using Back-propagation Neural Network, J Med Signals Sens, 6(3),2016, pp.172-82.

[S56] H.Ogihara, N.Iizuka, Y.Hamamoto, Prediction of Early Recurrence of Liver Cancer by a Novel Discrete Bayes Decision Rule for Personalized Medicine, BioMed research international, 2016(1),2016.

[S57] M. Dorado-Moreno, M.Pérez-Ortiz, PA.Gutiérrez, R.Ciria, J.Briceño, C.Hervás-Martínez , Dynamically weighted evolutionary ordinal neural network for solving an imbalanced liver transplantation problem, Artif Intell Med, 77(2017), pp.1-11.

[S58] L.Lau, Y.Kankanige, B.Rubinstein, R.Jones, C.Christophi, V.Muralidharan, J.Bailey, Machine-Learning Algorithms Predict Graft Failure After Liver Transplantation, Transplantation, 101(4),2017, pp.e125-e132.

[S59] MF.Byrne, N.Chapados, F.Soudan, C.Oertel, M.Linares Pérez, R.Kelly, N.Iqbal, F.Chandelier, DK.Rex , Real-time differentiation of adenomatous and hyperplastic diminutive colorectal polyps during analysis of unaltered videos of standard colonoscopy using a deep learning model, Gut, 2017.

[S60] T.Zhou, G.Han, BN.Li, Z.Lin, EJ.Ciaccio, PH.Green, J.Qin, Quantitative analysis of patients with celiac disease by video capsule endoscopy: A deep learning method, Comput Biol Med, 85(2017).

[S61] Y.Yuan, MQ.Meng , Deep learning for polyp recognition in wireless capsule endoscopy images, Med Phys, 44(4),2017.

[S62] G.Urban, P.Tripathi, T.Alkayali, M.Mittal, F.Jalali, W.Karnes, P.Baldi, Deep Learning Localizes and Identifies Polyps in Real Time With 96% Accuracy in Screening Colonoscopy, Gastroenterology, 155(4),2017.

[S63] Y.Chen, Y.Luo, W.Huang, D.Hu, RQ.Zheng, SZ.Cong, FK.Meng, H.Yang, HJ.Lin, Y.Sun, XY.Wang, T.Wu, J.Ren, SF.Pei, Y.Zheng, Y.He, Y.Hu, N.Yang, H.Yan , Machine-learning-based classification of real-time tissue elastography for hepatic fibrosis in patients with chronic hepatitis B, Comput Biol Med, 89(2017), pp.18-23.

[S64] J.Lara, M.Teka, Y.Khudyakov, Identification of recent cases of hepatitis C virus infection using physical-chemical properties of hypervariable region 1 and a radial basis function neural network classifier, BMC Genomics, 18(Suppl 10),2017, pp.33-42.

[S65] VV.Chirikov, FT.Shaya, E.Onukwugha, CD.Mullins, S.dosReis, CD.Howell , Tree-based Claims Algorithm for Measuring Pretreatment Quality of Care in Medicare Disabled Hepatitis C Patients, Med Care, 55(12),2017, pp.e104-e112.

[S66] K.Men, J.Dai, Y.Li, Automatic segmentation of the clinical target volume and organs at risk in the planning CT for rectal cancer using deep dilated convolutional neural networks, Med Phys, 44(12),2017, pp.6377-6389.

[S67] H.Haj-Hassan, A.Chaddad, Y.Harkouss, C.Desrosiers, M.Toews, C.Tanougast, Classifications of Multispectral Colorectal Cancer Tissues Using Convolution Neural Network, J Pathol Inform,2017.

[S68] J.Liu, D.Wang, L.Lu, Z.Wei, L.Kim, EB.Turkbey, B.Sahiner, NA.Petrick, RM.Summers , Detection and diagnosis of colitis on computed tomography using deep convolutional neural networks, Med Phys, 44(9),2017, pp.4630-4642.

[S69] S.Liu, M.Xu, J.Yang, H.Qi, F.He, X.Zhao, P.Zhou, L.Zhang, D.Ming , Research on Gastroesophageal Reflux Disease Based on Dynamic Features of Ambulatory 24-Hour Esophageal pH Monitoring, Comput Math Methods Med, 2017(1),2017.

[S70] V.Sehgal, A.Rosenfeld, DG.Graham, G.Lipman, R.Bisschops, K.Ragunath, M.Rodriguez-Justo, M.Novelli, MR.Banks, RJ.Haidry, LB.Lovat , Machine Learning Creates a Simple Endoscopic Classification System that Improves Dysplasia Detection in Barrett's Oesophagus amongst Non-expert Endoscopists, Gastroenterol Res Pract, 2018(1),2018.

[S71] R1.Ogawa, J.Nishikawa, E1.Hideura, A.Goto, Y.Koto, S.Ito, M.Unno, Y.Yamaoka, R.Kawasato, S.Hashimoto, T.Okamoto, H.Ogihara, Y.Hamamoto, I.Sakaida , Objective Assessment of the Utility of Chromoendoscopy with a Support Vector Machine, J Gastrointest Cancer,2018.

[S72] N.Ito, H.Kawahira, H.Nakashima, M.Uesato, H.Miyauchi, H.Matsubara , Endoscopic Diagnostic Support System for cT1b Colorectal Cancer Using Deep Learning, Oncology ,2018, pp.1-7.

[S73] HI.Shousha, AH.Awad, DA.Omran, MM.Elnegouly, M.Mabrouk, Data Mining and Machine Learning Algorithms Using IL28B Genotype and Biochemical Markers Best Predicted Advanced Liver Fibrosis in Chronic Hepatitis C, Jpn J Infect Dis, 71(1),2018, pp.51-57.

[S74] M.Nilashi, H. Ahmadi, L.Shahmoradi, O.Ibrahim, E.Akbari, A predictive method for hepatitis disease diagnosis using ensembles of neuro-fuzzy technique, J Infect Public Health,2 018.

[S75] H.Takiyama, T.Ozawa, S.Ishihara, M.Fujishiro, S.Shichijo, S.Nomura, M.Miura, T.Tada, Automatic anatomical classification of esophagogastroduodenoscopy images using deep convolutional neural networks, Scientific reports, 8:7497, 2018.

[S76] JY.He, X.Wu, YG.Jiang, Q.Peng, R.Jain , Hookworm Detection in Wireless Capsule Endoscopy Images With Deep Learning, IEEE Trans Image Process, 27(5),2018, pp.2379-2392.

[S77] K.Chaudhary, OB.Poirion , L.Lu, LX.Garmire, Deep Learning-Based Multi-Omics Integration Robustly Predicts Survival in Liver Cancer, Clin Cancer Res, 24(6),2018, pp.1248-1259.

[S78] Y.Horie, T.Yoshio, K.Aoyama, S.Yoshimizu, Y.Horiuchi, A.Ishiyama, T.Hirasawa, T.Tsuchida, T.Ozawa, S.Ishihara, Y.Kumagai, M.Fujishiro, I.Maetani, J.Fujisaki, T.Tada, Diagnostic outcomes of esophageal cancer by artificial intelligence using convolutional neural networks, Gastrointest Endosc, 2018.

[S79] S.Li, H.Jiang, Z.Wang, G.Zhang, YD.Yao , An effective computer aided diagnosis model for pancreas cancer on PET/CT images, Comput Methods Programs Biomed, 165(2018), pp.205-214.

[S80] T.Itoh, H.Kawahira, H. Nakashima, N.Yata, Deep learning analyzes Helicobacter pylori infection by upper gastrointestinal endoscopy images, Endosc Int Open, 6(2),2018, pp.E139-E144.

[S81] I.Morilla, M.Uzzan, D.Laharie, D.Cazals-Hatem, Q.Denost, F.Daniel, G.Belleannee, Y.Bouhnik, G.Wainrib, Y.Panis, E.Ogier-Denis, X.Treton, Colonic microRNA Profiles, Identified by a Deep-learning Algorithm, That Predict Responses of Patients With Acute Severe Ulcerative Colitis to Therapy, Clin Gastroenterol Hepatol,2018.

[S82] KJ.Choi, JK.Jang, SS.Lee, YS.Sung, WH.Shim, HS.Kim, J.Yun, JY.Choi, Y.Lee, BK.Kang, JH.Kim, SY.Kim, ES.Yu , Development and Validation of a Deep Learning System for Staging Liver Fibrosis by Using Contrast Agent-enhanced CT Images in the Liver, Radiology, 289(3),2018, pp.688-697.

[S83] K. Yasaka, H.Akai, O.Abe, S.Kiryu, Deep Learning with Convolutional Neural Network for Differentiation of Liver Masses at Dynamic Contrast-enhanced CT: A Preliminary Study, Radiology, 286(3),2018, pp.887-896.

[S84] J.Woolsey, J.Cárdenas-Rodríguez, J.Lee, A.Burkett, R.Lee Korn, Prediction of clinical outcomes for early gastric cancer using radiomic signatures derived from the quantitative texture analysis of conventional CT scans and machine learning, Journal of Clinical Oncology, 36(15_suppl),2018.

[S85] S.Moccia, LS.Mattos, I.Patrini, M.Ruperti, N.Poté, F.Dondero, F.Cauchy, A.Sepulveda, O.Soubrane, E.De Momi, A.Diaspro, M.Cesaretti , Computer-assisted liver graft steatosis assessment via learning-based texture analysis, Int J Comput Assist Radiol Surg, 13(9),2018, pp.1357-1367.

[S86] YZ.Zeng, SH.Liao, P.Tang, YQ.Zhao, M.Liao, Y.Chen, YX.Liang , Automatic liver vessel segmentation using 3D region growing and hybrid active contour model, Comput Biol Med, 2018.

[S87] Y.Yuan, B.Li, MQ.Meng , Bleeding Frame and Region Detection in the Wireless Capsule Endoscopy Video, IEEE J Biomed Health Inform, 20(2),2018, pp.624-630.

[S88] M.Biswas, V.Kuppili, DR.Edla, HS.Suri, L.Saba, RT.Marinhoe, JM.Sanches, JS.Suri , Symtosis: A liver ultrasound tissue characterization and risk stratification in optimized deep learning paradigm, Comput Methods Programs Biomed, 155(2018), pp.165-177.
